# Effects of *IGFBP4* deficiency on human preadipocyte proliferation and differentiation through the IGF1R/AKT pathway

**DOI:** 10.1002/2211-5463.70282

**Published:** 2026-06-08

**Authors:** Yujia Guo, Sandy Richter, Christoph Bach, Wieland Kiess, Anna Kirstein, Diana Le Duc, Antje Garten

**Affiliations:** ^1^ Center for Pediatric Research University Hospital for Children & Adolescents, Leipzig University Germany; ^2^ Department of Neurosurgery Leipzig University Germany; ^3^ LIFE Leipzig Research Centre for Civilization Diseases Leipzig University Germany; ^4^ German Center for Child and Adolescent Health (DZKJ), DZKJ site Leipzig, Dresden Germany; ^5^ Institute of Human Genetics Leipzig University Medical Center Germany; ^6^ Institute for Clinical Genetics University Clinics Carl‐Gustav Carus, Technical University of Dresden Germany

**Keywords:** adipogenesis, human preadipocytes, IGF1 receptor (IGF1R), IGF1R protein degradation, IGF1R/AKT pathway, IGFBP4

## Abstract

Adipogenesis involves preadipocyte proliferation and differentiation into mature adipocytes, with dysregulation of this process being associated with changes in metabolic health. Here, we aimed to evaluate the effect of *IGFBP4* knockdown (KD) on human preadipocytes and define if this altered IGF1 signaling. We assessed proliferation, adipogenic differentiation, gene expression, and the activation of the IGF1 receptor (IGF1R)/AKT pathway across different human adipocyte models. Our findings show *IGFBP4* KD impaired proliferation and caused a reduction in IGF1R and phosphorylated AKT (Ser473) proteins, although no difference in adipogenic differentiation was observed. Furthermore, while adding recombinant human IGFBP4 did not reverse this effect, protein synthesis inhibition abolished *IGFBP4* KD‐mediated IGF1R downregulation when cotreated with a proteasomal inhibitor but not with a lysosomal inhibitor. We conclude that *IGFBP4* knockdown is associated with IGF1R downregulation, impaired AKT phosphorylation, and suppressed proliferation, suggesting IGFBP4 is a critical regulator of IGF1R homeostasis in human preadipocytes.

## Graphical abstract

*IGFBP4* knockdown (KD) impairs preadipocyte proliferation and is associated with IGF1R protein downregulation and attenuated AKT phosphorylation. The mechanisms by which *IGFBP4* KD influences the IGF1R/AKT signaling pathway involve newly synthesized proteins and lysosomal degradation pathways. Created in BioRender.

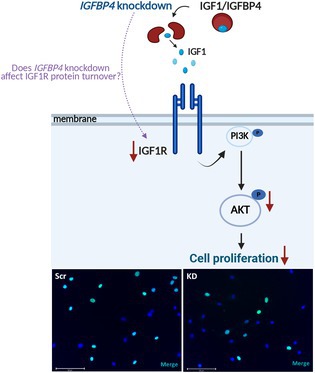

**Abbreviations**
AGPAT2: 1‐acylglycerol‐3‐phosphate acyltransferase 2
AKT: protein kinase B (or PKB)
ALS: acid‐labile subunit
C/EBP*α*
: CCAAT/enhancer‐binding protein alpha
CHX: cycloheximide
DMEM: Dulbecco's Modified Eagle's Medium
EdU: 5‐ethinyl‐2′‐deoxyuridine
EGF: epidermal growth factor
EGFR: epidermal growth factor receptor
HPRT: hypoxanthine phosphoribosyltransferase
IGF1: insulin‐like growth factor 1
IGF1R: IGF1 receptor
IGFBP4: insulin‐like growth factor binding protein 4
IGFBPs: insulin‐like growth factor binding proteins
INSR: insulin receptor
KD: *IGFBP4* knockdown
OF: serum‐free medium
OF+IGF1: serum‐free medium supplemented with IGF1
PAPP‐A: pregnancy‐associated plasma protein‐A
PHTS: phosphatase and tensin homolog hamartoma tumor syndrome
PI3K: phosphoinositide 3‐kinase
PPAR‐γ: peroxisome proliferator‐activated receptor gamma
RIPA: radioimmunoprecipitation assay
Scr: control cells transfected by scrambled RNA
SGBS: Simpson‐Golabi‐Behmel Syndrome
siRNA: small interfering RNA
SREBP1: sterol regulatory element‐binding protein 1
SVF: stromal‐vascular fraction
TBP: TATA‐box binding protein
TBST: TBS buffer containing 0.1% Tween 20

Adipogenesis is the process through which mesenchymal stem cells commit to the adipocyte lineage, and preadipocytes differentiate to mature adipocytes [1]. It is fundamental to adipose tissue development and expansion. Dysregulation of adipogenesis is intricately linked to metabolic health. Understanding the intrinsic molecular networks governing adipocyte commitment, proliferation, and terminal differentiation is therefore crucial for identifying novel therapeutic targets for adipose tissue‐related diseases.

Insulin‐like growth factor 1 (IGF1) is central to the regulatory network of adipogenesis. As an anabolic factor, IGF1 modulates adipocyte development and function by enhancing preadipocyte replication and differentiation. This effect is evident in models ranging from murine 3 T3‐L1 and primary stromal‐vascular fraction (SVF) cell cultures to human mesenchymal stem cell lines [2, 3]. IGF1 is an essential extracellular proliferative trigger, by stimulating cells to progress through G1 into S phase of the cell cycle [4]. It can also promote adipocyte differentiation. Nanomolar concentrations of IGF1 are sufficient to induce differentiation and lipid accumulation in human mesenchymal stem cells, in place of insulin at micromolar concentrations [3]. IGF1 acts synergistically with glucocorticoids and cAMP‐elevating agents as a differentiation inducer to commit cells to terminal differentiation [5].

Actions of IGF1 are mediated by binding to insulin‐like growth factor 1 receptor (IGF1R), which is present in a wide range of cell types [6, 7]. The bioavailability and activity of IGF1 are tightly modulated by insulin‐like growth factor binding proteins (IGFBPs) [8]. Among them, IGFBP4 is a high‐affinity binder that predominantly functions as an inhibitor of IGF1 action by sequestering IGF1 and preventing its binding to the IGF1R, thereby attenuating downstream signaling cascades like the PI3K/AKT pathway, which are critical for both cell proliferation and adipogenic differentiation [6, 8].

Notably, IGFBP4 is expressed in adipocytes [9], and studies in *Igfbp4* knockout mice suggest its involvement in modulating IGF1‐mediated adipose tissue expansion, highlighting its potential role as a physiological regulator of adipogenesis *in vivo* [10]. Intriguingly, emerging evidence indicates that IGFBP4 may also exert biological effects independent of IGF1 binding. For instance, IGFBP4 has been shown to inhibit the canonical Wnt/β‐catenin signaling pathway, a pathway known to potently suppress adipocyte commitment and early differentiation during processes such as cardiogenesis [11]. This suggests a potential IGF1‐independent, modulatory role of IGFBP4 on signaling pathways relevant to adipocyte lineage determination.

Despite its expression in adipose tissue and well‐characterized role as an IGF‐binding protein, the specific function of IGFBP4 in human adipogenesis remains poorly defined. In particular, the mechanisms by which IGFBP4 regulates human preadipocyte proliferation and differentiation, through the IGF1R signaling pathway, remain unclear.

Based on this background and the identified knowledge gap, we hypothesize that IGFBP4 is a key regulator of human adipogenesis, primarily through modulating IGF1 signaling, and that *IGFBP4* deficiency will impact both preadipocyte proliferation and differentiation. To test this hypothesis, we used human preadipocyte models to investigate the functional impact of *IGFBP4* knockdown on preadipocyte proliferation and adipocyte differentiation, including lipid accumulation and expression of key adipogenic markers. Furthermore, we explored the potential molecular mechanisms underlying these effects, with a focus on how *IGFBP4* knockdown influences the IGF1R and downstream AKT signaling pathway in the context of human adipogenesis.

## Methods

### Cell culture

LipPD1 cells were obtained from resected lipoma tissue from an 11‐month‐old male patient with phosphatase and tensin homolog hamartoma tumor syndrome (PHTS) caused by a heterozygous germline *PTEN* deletion [12]. SGBS cells were a kind gift from Prof. M. Wabitsch, Ulm University, and cultured and differentiated as previously described [13]. Primary human stromal vascular fraction cells (SVF) were isolated from visceral or subcutaneous adipose tissue of healthy donors, resected during bariatric surgery. The study was approved by the Leipzig University ethics committee (ethics approval: no. 425–12‐171 220) and has been performed in accordance with the principles laid down in the 1964 Declaration of Helsinki and its later amendments. All adipose tissue and lipoma tissue donors gave written informed consent to participate in the study.

Isolation and culture methods were described previously [14]. Briefly, cells were cultured in Dulbecco's Modified Eagle's Medium (DMEM)/F12 medium supplemented with 10% fetal bovine serum (# S0615; Sigma), glutamine (2 mm), biotin (# B4639; Sigma, 33 mm), and pantothenic acid (# P5155; Sigma, 17 mm) at 37 °C containing 5% CO_2_. Cells were passaged every 3 or 4 days.

### Small interfering RNA (siRNA)‐mediated knockdown (KD) of *IGFBP4*

The day before transfection, LipPD1 cells were seeded at a density of 200 000–250 000 cells per 15‐cm^2^ plate for optimal growing conditions. Transfection was performed using the Neon NxT Electroporation System 100 μL Kit (Invitrogen; Thermo Fisher Scientific, Waltham, MA, USA). Three *IGFBP4* siRNAs (Silencer Select siRNA IGFBP4 s7230, s7231, s7232; # ASO2NRQB; Invitrogen, Thermo Fisher Scientific) (final concentration: 20 nm) or scrambled RNA (Silencer Negative Control siRNA # 1; Ambion, Thermo Scientific, Waltham, MA, USA, # AM4635) were added to cell pellets, which were then resuspended in 100 ul gene editing buffer (Thermo Fisher Scientific) for electroporation in Neon NxT 100 ul tips at 1300 volts, 2 pulses, and 20‐ms width. Transfected cells were transferred to prewarmed culture medium and seeded on multiwell plates. Medium change to culture medium, differentiation medium, or serum‐free medium (according to different experiments) was performed 24 h after transfection. The efficiency of *IGFBP4* KD was quantified by qPCR and immunoblotting.

### LipPD1 preadipocyte proliferation

After transfection, cells were seeded at a density of 2000 cells/well on 96‐well plates for proliferation, and 150 000 cells/well on 6‐well plates in culture medium for measuring mRNA and protein.

To assess the activation of the IGF1R/AKT signaling pathway, the medium was changed to culture medium or serum‐free medium (OF) after 24 h (Day 0) and incubated for another 24 h. On Day 1, IGF1 (100 nm) was added to OF medium for 15 min before harvesting cells.

For cell proliferation assay, 24 h post transfection (Day 0), the medium was changed to culture medium, OF medium, or serum‐free medium supplemented with IGF1 (100 nm) (OF+IGF1) for 6 days' incubation. Cells were fixed on Day 0 and Day 6, and Hoechst 33342 (1 μg/mL, Sigma) staining was used to stain cell nuclei for 5 min. Images were taken using the EVOS FL Auto 2 Cell Imaging System (Invitrogen; Thermo Fisher Scientific) with the DAPI channel. Cell counting was performed with ImageJ, and the results were compared between two time points.

To monitor cell growth, the confluence of cells was measured every 4 h for 5 days by live cell imaging using an IncuCyte SX5 (Sartorius, Göttingen, Germany), starting 6 h after knockdown and subsequent medium change.

### Ki67 staining

Ki67 is used as a proliferation marker to determine the growth fraction of a cell population. For Ki67 immunofluorescence staining, transfected cells were seeded at a density of 2000 cells/well on 96‐well plates and fixed with 4% Roti‐Histofix (# P087.4; Roth, Karlsruhe, Germany) after 24 h (Day 0). Cells were blocked in IF buffer (DPBS + 5% BSA + 0.3% Tween) for 1 h at room temperature and incubated with Ki67 primary antibody (Dako, Glostrup, Denmark, M7240, 1 : 200 dilution in IF buffer) overnight at 4 °C. The other day, cells were washed with IF buffer three times for 5 min and incubated with secondary Alexa Fluor 488 antibody (Invitrogen, A11001, 1 : 1000 dilution in IF buffer) for 2 h at RT in the dark. Followed by washing with IF buffer one time and incubating with Hoechst 33342 (1 μg/mL, Sigma, # 14533) in DPBS for 5 min. Microscope images were taken with the EVOS FL Auto 2 Cell Imaging System (Invitrogen; Thermo Fisher Scientific), and cell counting was performed with the Celleste Image Analysis Software (Thermo Fisher Scientific). The proliferation index was calculated as: Ki67‐positive nuclei/total nuclei × 100%.

### 5‐ethynyl‐2′‐deoxyuridine (EdU) cell proliferation assay

EdU (EdU‐Click 488, baseclick GmbH, Neuried, Germany) was used according to manufacturer's instructions for labeling newly synthesized DNA. Briefly, after *IGFBP4* knockdown, cells were seeded at a density of 2000 cells/well on 96‐well plates overnight for attachment. EdU‐labeling and detection were performed 24 h later (day 0): Cells were incubated with 10 μm EdU for 2 h at 37 °C, followed by fixation with 4% Roti‐Histofix PFA (# P087.4; Roth), washed twice with 3% BSA in PBS, and permeabilized with 0.5% Triton X‐100 (Sigma) for 20 min at room temperature. For EdU detection, reaction cocktail containing 6‐FAM‐Azide was added after washing twice with PBS‐3% BSA and cells were incubated for 30 min at room temperature in the dark. After washing steps, nuclei were stained with Hoechst 33342 (# 14533; Sigma, 1 μg/mL) for 5 min. Images were taken by EVOS FL Auto 2 Cell Imaging System (Invitrogen; Thermo Fisher Scientific) and counting for EdU‐labeled cell nuclei over total cell nuclei was performed with the Celleste Image Analysis Software (Thermo Fisher Scientific).

### Cellular senescence

To exclude the possibility of cell senescence after *IGFBP4* knockdown, beta‐galactosidase staining was performed (β‐Galactosidase kit # 9860, Cell Signaling, Danvers, MA, USA).

After transfection, cells were seeded at a density of 2000 cells/well on 96‐well plates for attachment overnight and were fixed 24 h later (Day 0). Hoechst 33342 (1 μg/mL, Sigma) staining was used to stain cell nuclei. β‐galactosidase staining solution was added to both scr control and *IGFBP4* KD cells. Plates were sealed with parafilm to prevent evaporation and incubated overnight at 37 °C in a dry incubator (without CO_2_). Microscope images were taken with the EVOS FL Auto 2 Cell Imaging System (Invitrogen; Thermo Fisher Scientific).

### LipPD1 preadipocyte differentiation

For differentiation, after transfection, cells were seeded at a density of 15 000 cells/well on 96‐well plates or 150 000 cells/well on 6‐well plates in culture medium. The medium was changed to differentiation medium [14] (Dulbecco's Modified Eagle's Medium (DMEM)/F12 medium contains glutamine (2 nm), biotin (8 mg/mL), pantothenic acid (4 mg/L), human apotransferrin (0.01 mg/mL), human insulin (20 nm), hydrocortisone (100 nm), triiodo L thyronine (0.2 nm), dexamethasone (25 nm), isobutyl‐1‐methyl xanthine (500 μm), and rosiglitazone (2 μm)) 24 h later (Day 0). Cells in 96‐well plates were differentiated for 9 days before fixation and evaluating lipid accumulation, while cells in 6‐well plates were differentiated for 3 days and 5 days before harvesting cell lysates for RNA isolation and protein extraction.

### Nile red/Hoechst staining

Adipogenesis was evaluated by measuring the percentage of lipid accumulating cells after adipogenic differentiation. After 9 days of differentiation, cells were fixed with 4% Roti‐Histofix PFA (# P087.4; Roth) and then stained with the fluorescent dyes Nile Red (# N3013; Sigma, 0.5 μg/mL) and Hoechst 33342 (# 14533; Sigma, 1 μg/mL) for 5 min in DPBS. Microscope images were taken with the EVOS FL Auto 2 Cell Imaging System (Invitrogen; Thermo Fisher Scientific) by randomly choosing areas per well. The counting for total cell number and lipid‐accumulating cells was performed with the Celleste Image Analysis Software (Thermo Fisher Scientific).

### RNA isolation, reverse transcription, quantitative polymerase chain reaction (RT–qPCR)

mRNA was isolated from LipPD1 cells using the RNeasy Mini Kit (Qiagen GmbH, Hilden, Germany) according to the manufacturer's protocol. 500 ng mRNA was reverse transcribed into cDNA. Quantitative PCR was performed using Taqman Master Mix Plus Low ROX (Eurogentec, Liege, Belgium) or SYBR‐Green Low ROX Mix (Thermo Fisher Scientific, Inc.) and the Applied Biosystems Real‐Time PCR System (Thermo Fisher Scientific, Inc.).

The copy number of each sample was calculated from the standard curve and normalized to the housekeeping genes, *TATA‐box binding protein* (*TBP*) and/or *hypoxanthine phosphoribosyltransferase* (*HPRT*) expressions. Primers sequences for targeted genes were listed in Table 1.

**Table 1:** Primers used for RT‐qPCR.

Target	Forward	Reverse	Probe
*hTBP* (NM_003194.5)	TTG TAA ACT TGA CCT AAA GAC CAT TGC	TTC GTG GCT CTC TTA TCC TCA TG	AAC GCC GAA TAT AAT CCC AAG CGG TTT G
*hHPRT* (NM_000194.3)	GGC AGT ATA ATC CAA AGA TGG TCA A	GTC TGG CTT ATA TCC AAC ACT TCG T	CAA GCT TGC TGG TGA AAA GGA CCC C
*hIGFBP4* (NM_001552)	ACCCACGAGGACCTCTACATCA	CACACCAGCACTTGCCACGCT	
*hIGF1R* (NM_000875.5)	CTGGCCGACGAGTGGAG	TGGAGGTAGCCCTCGATCA	
*hIGF1* (NM_001111283.3)	GCTCTTCAGTTCGTGTGTGGA	GCCTCCTTAGATCACAGCTCC	
*hINSR* (NM_000208.4)	CGGAACCCACCTATTTCTACG	AGATGAGGGGGCCGATGATA	
*hPPAR‐γ* (NM_001354668.2)	GATCCAGTGGTTGCAGATTACAA	GAGGGAGTTGGAAGGCTCTTC	TGACCTGAAACTTCAAGAGTACCAAAGTGCAA
*hAKT1* (NM_001382430.1)	ATGCAGCATCGCTTCTTTGC	GGCCGTGAACTCCTCATCAA	
*hAKT2* (NM_001626.6)	GTCCCGAGCTAGGTGACAGC	TGGCCTCCAGGTCTTGATGTA	

### Western blot analysis

For protein extraction, cells were lysed with modified radioimmunoprecipitation assay (RIPA) buffer (supplemented with Na‐vanadat, NaF, Roche Complete). Protein concentrations were determined by BCA assay (Thermo Fisher Scientific, Inc.) using CLARIOstar (BMG LABTECH). 15 μg proteins were loaded in each lane of the SDS/PAGE gel, followed by semi‐dry transfer onto nitrocellulose membranes, blocking with 5% non‐fat dry milk in TBS buffer containing 0.1% Tween 20 (TBST) for 1 h at room temperature, washing three times for 5 min with TBST, and overnight incubation with the appropriate primary and secondary antibodies as listed in Table 2. *α*‐tubulin was used as the loading control. Western blots for AKT and *α*‐tubulin were subsequently stripped after phosphorylated AKT (Ser473) and re‐immunoblotted with correspondent antibodies. Protein bands were detected using Amersham ECL Prime Western Blotting Detection Reagent (Cytiva). Densitometric analysis was performed using ImageJ [15].

**Table 2:** Antibodies used for western blot (WB) and immunofluorescence staining (IF).

	Dilution	Supplier	Cat. no.
Primary antibody			
IGFBP4 (A3) Mouse mAb sc‐517 440	1 : 500	Santa Cruz	#B0824
IGF1R beta Rabbit Ab	1 : 1000	CST	#3027 L, #3027S
Phospho‐AKT (Ser473) (D9E) Rabbit mAb	1 : 1000	CST	#4060S
AKT antibody Rabbit Ab	1 : 1000	CST	#9272S
*α*‐tubulin (11H10) Rabbit mAb	1 : 1000	CST	#2125
Ki67 (MIB‐1) Mouse mAb	1 : 200 IF‐buffer	Dako	M7240
Secondary antibody			
Horseradish peroxidase (HRP)‐goat anti rabbit	1 : 2000	Dako	P0448
Horseradish peroxidase (HRP)‐goat anti mouse	1 : 1000	Dako	P0447
Alexa Fluor 488 goat anti‐mouse IgG H + L	1 : 1000 IF‐buffer (IF)	Invitrogen	A11001

### Addition of recombinant IGFBP4 after *IGFBP4* KD

After *IGFBP4* knockdown, cells were seeded at a density of 150 000 cells/well on 6‐well plates in culture medium. Recombinant human IGFBP4 40 nm (PeproTech or Hycultec GmbH) was added to both the control‐transfected and knocked‐down cells directly after KD. Cells were then incubated for 48 h.

### Addition of recombinant human IGF1

Cells were seeded at a density of 150 000 cells/well on 6‐well plates in culture medium overnight for attachment. Culture medium was changed 24 h later, and IGF1 (# IU100; GroPep, 1 μm, Thebarton, Australia) was added. Cells were then incubated for another 24 h before harvesting for protein extraction.

### Addition of proteasome inhibitor bortezomib and lysosomal inhibitor chloroquine

After *IGFBP4* knockdown, cells were seeded at a density of 120 000–150 000 cells/well on 6‐well plates overnight in culture medium.

To inhibit protein neo‐synthesis, cells were incubated in culture medium with protein synthesis inhibitor cycloheximide (CHX, 10ug/ml, Sigma‐Merck, St. Louis, MO, USA) for 30 min, which was maintained for the entire experiment. Bortezomib (1 μm, dissolved in DMSO), chloroquine (50 μm, Sigma‐Merck), or solvent control (DMSO) was added to cells for 6 h before harvesting.

### Statistical analysis

Data from independent experiments were presented as the mean ± SD. Statistical significance was assessed by the graphpad Prism 10 software. A paired Student's *t*‐test or a ratio paired Student's *t*‐test was used for comparing two groups. One‐way ANOVA was used for testing differences among more than two groups. Two‐way ANOVA followed by Tukey's multiple comparisons test was applied to analyze the effects of two different variables between groups. Results were considered significant at *P* < 0.05.

## Results

### *IGFBP4* knockdown (KD) impaired cell proliferation

**Fig. 1:**
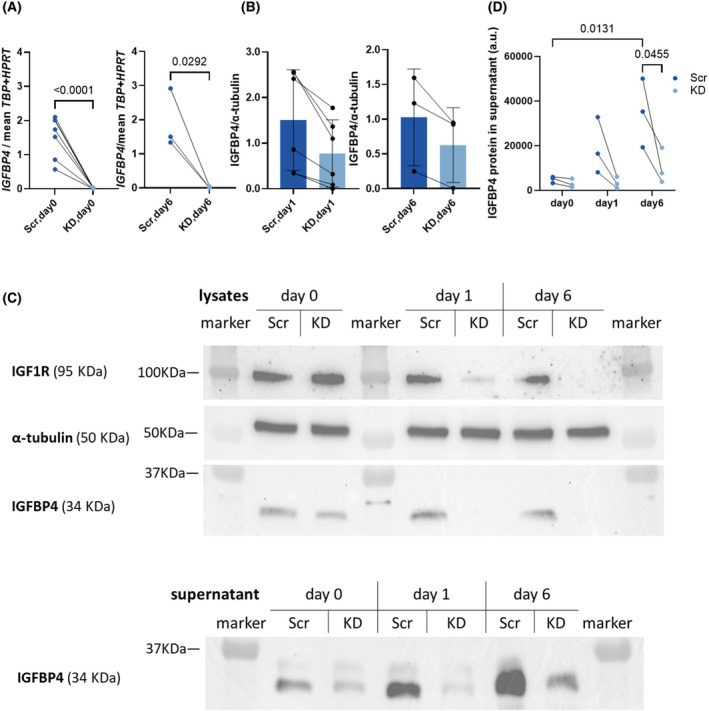
*IGFBP4* knockdown efficiency on mRNA and protein levels in cell lysates and supernatants. Scr represents control cells transfected by scrambled RNA; KD represents cells transfected with *IGFBP4* siRNAs. (A) *IGFBP4* KD efficiency in proliferating cells on mRNA level was assessed by quantitative PCR (qPCR) on day 0 (24 h after transfection) and day 6. *IGFBP4* mRNA level was decreased by 99.0% 24 h after transfection (*n* = 8, *P* < 0.0001, day 0) and by 99.2% 6 days after transfection (*n* = 3, *P* = 0.0292). Data were normalized to the average of *TATA‐box binding protein* (*TBP*) and *hypoxanthine phosphoribosyltransferase* (*HPRT*) and presented as mean ± SD. Significant differences were determined by ratio paired *t*‐test. (B) IGFBP4 protein KD efficiency in cell lysates, assessed by western blot. IGFBP4 protein decreased by 61% in *IGFBP4* KD cells compared to controls on day 1 (*n* = 6, 48 h after transfection) and by 54.3% on day 6 (*n* = 3). Data were normalized to *α*‐tubulin and quantified by densitometry. Data were presented as mean ± SD. (C) IGFBP4 protein in cell culture supernatant, assessed by western blot and quantified by densitometry on day 0 (24 h after transfection), day 1 (48 h after transfection) and day 6. IGFBP4 increased significantly in control supernatant from day 0 to day 6 (*P* = 0.0131). On day 6, IGFBP4 was significantly lower in the supernatant of *IGFBP4* KD cells compared to controls (*P* = 0.0455). Data were presented as mean ± SD from three independent experiments. Significant differences were determined by two‐way ANOVA. (D) Representative western blot images are shown.

Since IGFBP4 was described to negatively regulate IGF1‐mediated cellular processes by binding and sequestering IGF1, we tested the effect of *IGFBP4* KD on human preadipocyte proliferation. We first checked whether the efficiency of IGFBP4 downregulation would be similar throughout the duration of the 6‐day proliferation assay. *IGFBP4* mRNA level was decreased by 99.0% (*P* < 0.0001) 24 h after transfection (Day 0) and declined by 99.2% (*P* = 0.0292) 6 days after transfection (Fig. 1A). On the protein level, IGFBP4 protein declined by 61.0% in *IGFBP4* KD vs. control cells one day and by 54.3% 6 days after transfection (Fig. 1B,C). IGFBP4 protein increased during the experiment in supernatant of controls, but was consistently lower in *IGFBP4* KD supernatant. IGFBP4 protein decreased by 40.2%, 83.7%, and 70.1% on Day 0, Day 1, and Day 6, respectively (*P* = 0.0455 on Day 6, Fig. 1C,D). We also checked IGF1R expression and found that IGF1R was lower in *IGFBP4* KD cells on Day 1 (48 h after transfection) and Day 6 of the proliferation experiment, but not 24 h after transfection (Fig. 1C).

**Fig. 2:**
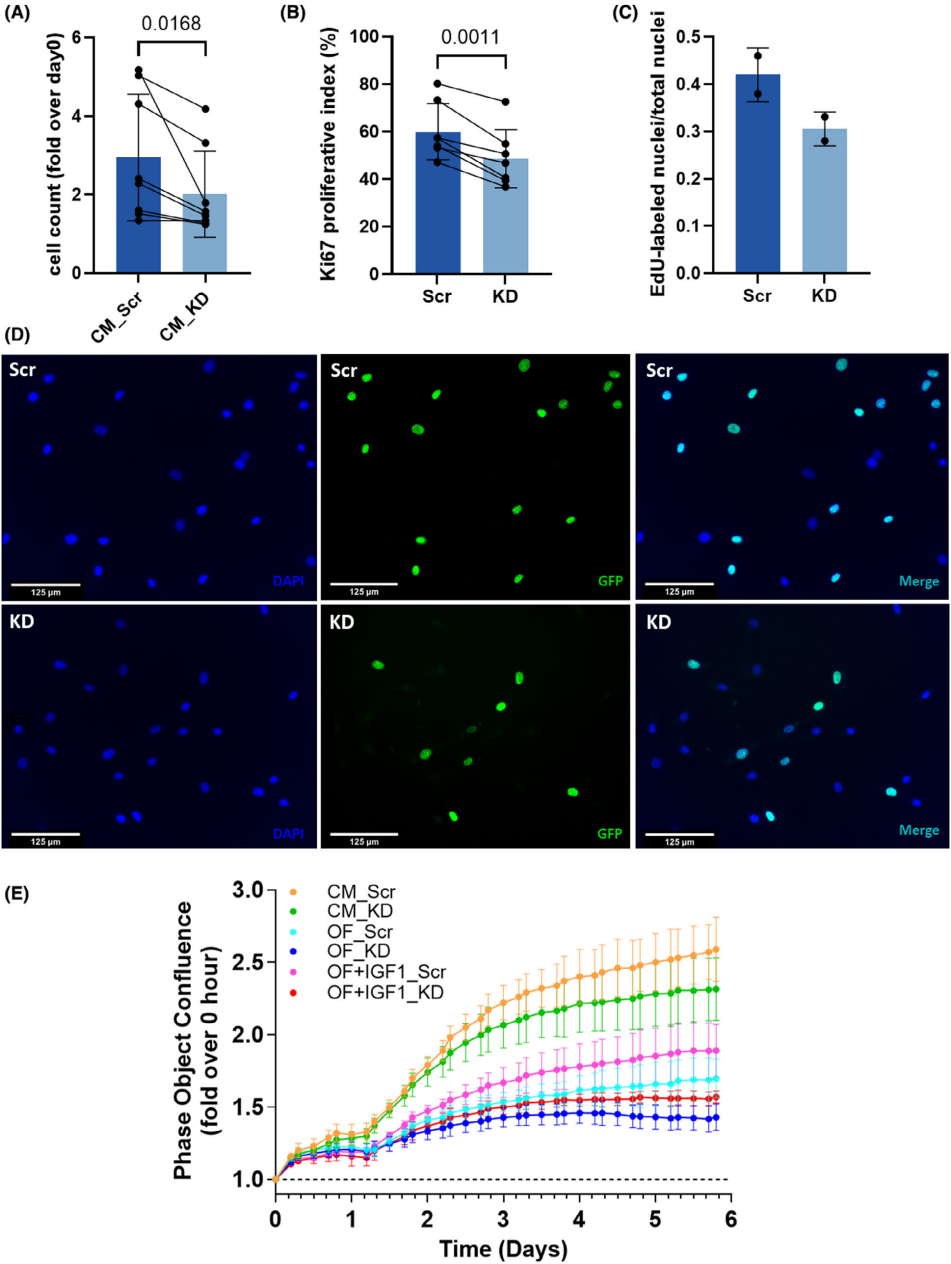
LipPD1 cells proliferation after *IGFBP4* knockdown. (A) Cell proliferation was assessed by cell counting. Cells were grown in culture medium (CM) for 6 days, and nuclei were stained with Hoechst. *IGFBP4* KD reduced cell proliferation to 0.68‐fold of control. Cell numbers were normalized to the mean of each Scr or KD group from day 0 and presented as mean ± SD (*n* = 8, *P* = 0.0168, by ratio paired *t*‐test). (B) Cell proliferative ability was assessed by Ki67 immunofluorescence staining. Cell proliferative index was determined as the percentage of Ki67‐positive cells, and it was decreased by 19.1% after *IGFBP4* KD (*n* = 7, *P* = 0.0011, by ratio paired *t*‐test). Data were presented as mean ± SD. (C) Analysis of DNA synthesis phase of the cell cycle by EdU incorporation. *IGFBP4* KD cells showed a decrease of EdU‐labeled nuclei on day 0 (24 h after transfection) compared to Scr control cells, corresponding to a 27% reduction (30.6 ± 3.6% in KD vs. 42.0 ± 5.7% in Scr controls). Data were presented as mean ± SD. (D) Representative images of fluorescence imaging of replicating LipPD1 cells detected with EdU. Blue signal/Left: DNA staining with Hoechst; Green signal/Middle: EdU labeling; Right: Merged Images. *n* = 2, scale bar = 125 μm. (E) Confluence (%) was measured by live cell imaging every 4 h. *IGFBP4* KD cells showed reduced growth in all growth conditions compared to Scr. Cells had different growth rates in different media, with those in the culture medium growing the fastest (Orange: Scr, in CM; green: KD, in CM; pink: Scr, in OF+IGF1; red: KD, in OF+IGF1; light blue: Scr, in OF; dark blue: KD, in OF). Data were presented as mean ± SD.

*IGFBP4* KD cells proliferated less than control cells after 6 days in culture medium (2.02 ± 1.11‐fold in KD vs. 2.96 ± 1.62‐fold in controls; *P* = 0.0168; Fig. 2A). We also tested the effect of IGF1 stimulation by adding 100 nm IGF1 to serum‐free medium but did not see a difference in cell counting after 6 days' incubation (Fig. S1A). On the day after transfection (Day 0), we counted slightly more cells in the *IGFBP4* KD group compared to control cells (Fig. S1B).

In support of the cell counting data, a decrease of proliferation marker Ki67‐positive nuclei was detected in *IGFBP4* KD cells versus controls on the day after knockdown in the proliferation assay (0.81‐fold of controls, 48.8 ± 12.3% in KD vs. 60.3 ± 11.9% in controls; *P* = 0.0011; Fig. 2B). Analysis of EdU incorporation into DNA showed that *IGFBP4* KD cells had a 27% reduction in DNA synthesis compared to control cells (30.6 ± 3.6% in KD vs. 42.0 ± 5.7% in controls; Fig. 2C,D).

When validating our results with live cell imaging, we observed that *IGFBP4* KD cells were less confluent than control cells under different culture conditions: in culture medium, in serum‐free medium, and in serum‐free medium with 100 nm IGF1 (Fig. 2E).

We checked whether senescence could have an influence on the difference in cell counting. After 6 days of incubation in culture medium, we found no senescent cells in controls and *IGFBP4* KD groups (Fig. S1C).

### No significant effect on adipocyte differentiation was observed after *IGFBP4* KD

**Fig. 3:**
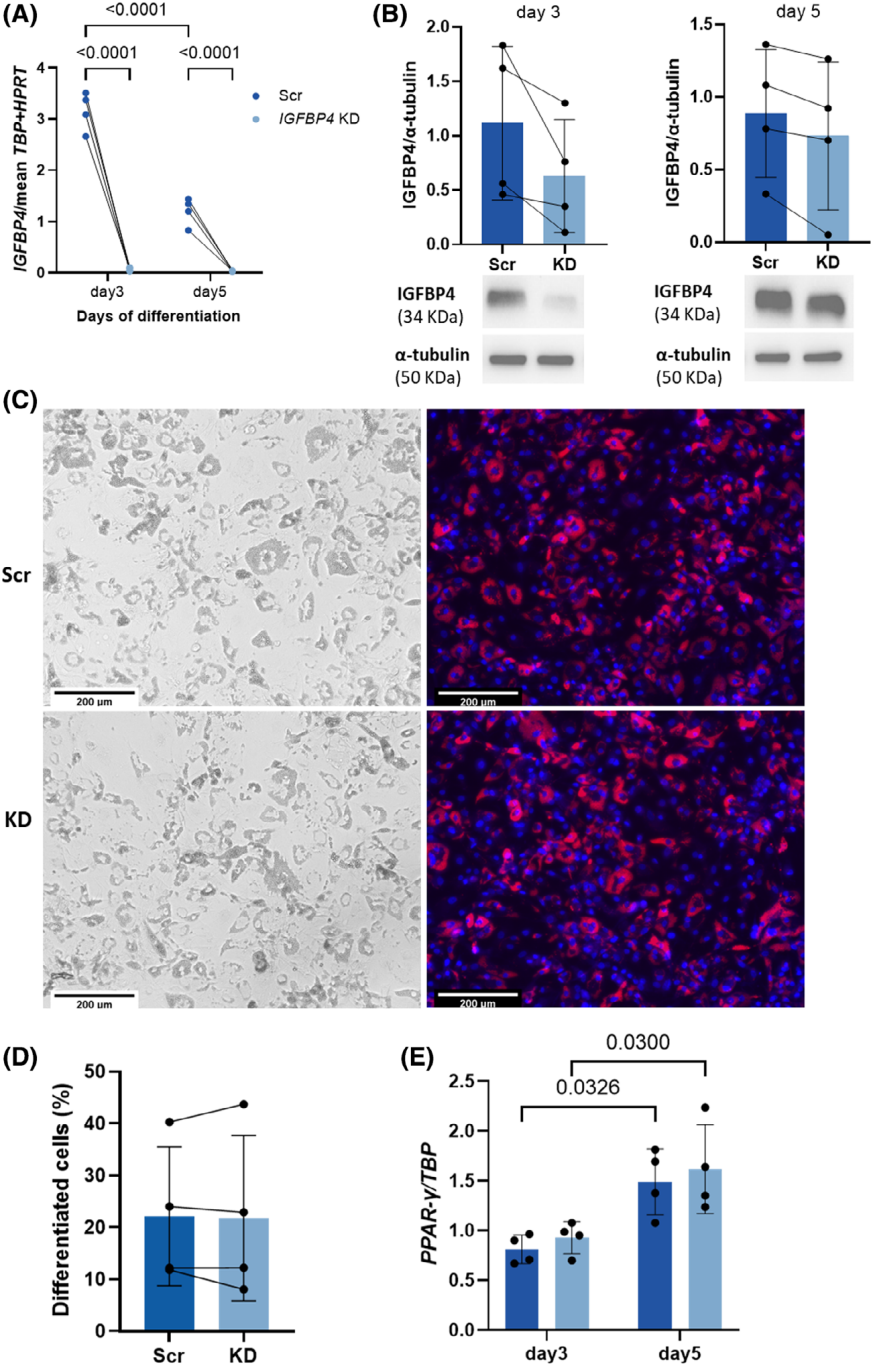
LipPD1 cells differentiation after *IGFBP4* knockdown. *IGFBP4* knockdown efficiency in differentiating cells on both mRNA (A) and protein levels (B). After *IGFBP4* KD, cells were differentiated for 3 and 5 days. (A) *IGFBP4* mRNA was assessed by qPCR. *IGFBP4* mRNA declined by approximately 97% to 98%. *IGFBP4* expression decreased by 62% during differentiation (comparing mRNA from Scr on day 5 to that from Scr on day 3. Data were normalized to the average of *TATA‐box binding protein* (*TBP*) and *hypoxanthine phosphoribosyltransferase* (*HPRT*) and presented as mean ± SD (*n* = 4, *P* < 0.0001, by two‐way ANOVA). (B) IGFBP4 protein was measured by western blot and quantified by densitometry. After knockdown, IGFBP4 proteins were downregulated by 30–46%. Data were normalized to *α*‐tubulin and presented as mean ± SD (*n* = 4, *P* > 0.05). (C) Microscopy images of lipid accumulation. Cells were fixed on the 9th day of differentiation and stained with Nile Red/Hoechst. Left: bright field; right: DAPI/RFP channels. Blue: nuclei; red: lipid droplets; *n* = 4, scale bar: 200 μm. (D) Differentiated cells calculation (%). Nile Red‐stained (lipids) cells were counted and normalized to cell nuclei numbers as determined by Hoechst staining. There was no significant decrease in lipid accumulation after knockdown. Data were presented as mean ± SD (*n* = 4). (E) Adipogenic marker *peroxisome proliferator‐activated receptor gamma* (*PPAR‐γ*) mRNA was not decreased after *IGFBP4* KD, but increased over the days of differentiation (*n* = 4, *P <* 0.05, by two‐way ANOVA). Data were presented as mean ± SD.

Since IGF1 is also implicated in adipocyte differentiation, we tested whether *IGFBP4* KD would influence *in vitro* differentiation of human preadipocytes. We found that *IGFBP4* was most highly expressed in early differentiated adipocytes (Fig. S2A) and therefore checked siRNA knockdown efficiency on Day 3, Day 5, and Day 9 of adipocyte differentiation. *IGFBP4* mRNA level was reduced by 97–98% (Fig. 3A) after knockdown, while IGFBP4 protein was downregulated by 30–46% (Fig. 3B). *IGFBP4* knockdown efficiency on mRNA level was assessed by qPCR and showed that *IGFBP4* mRNA was consistently downregulated on Day 3 and Day 9 (Fig. S2B). When checking IGFBP4 protein on Day 9 of differentiation, we found a slight increase in *IGFBP4* KD cell lysates compared to Day 3 (Fig. S2B). We did not observe a significant effect on lipid accumulation when comparing LipPD1 cells with transient *IGFBP4* KD to control cells after *in vitro* differentiation (Fig. 3C,D). The expression of adipogenic marker peroxisome proliferator‐activated receptor gamma (*PPAR‐γ*) was not significantly influenced by transient *IGFBP4* KD (Fig. 3E).

### *IGFBP4* KD led to a downregulation of IGF1R and phosphorylated AKT

**Fig. 4:**
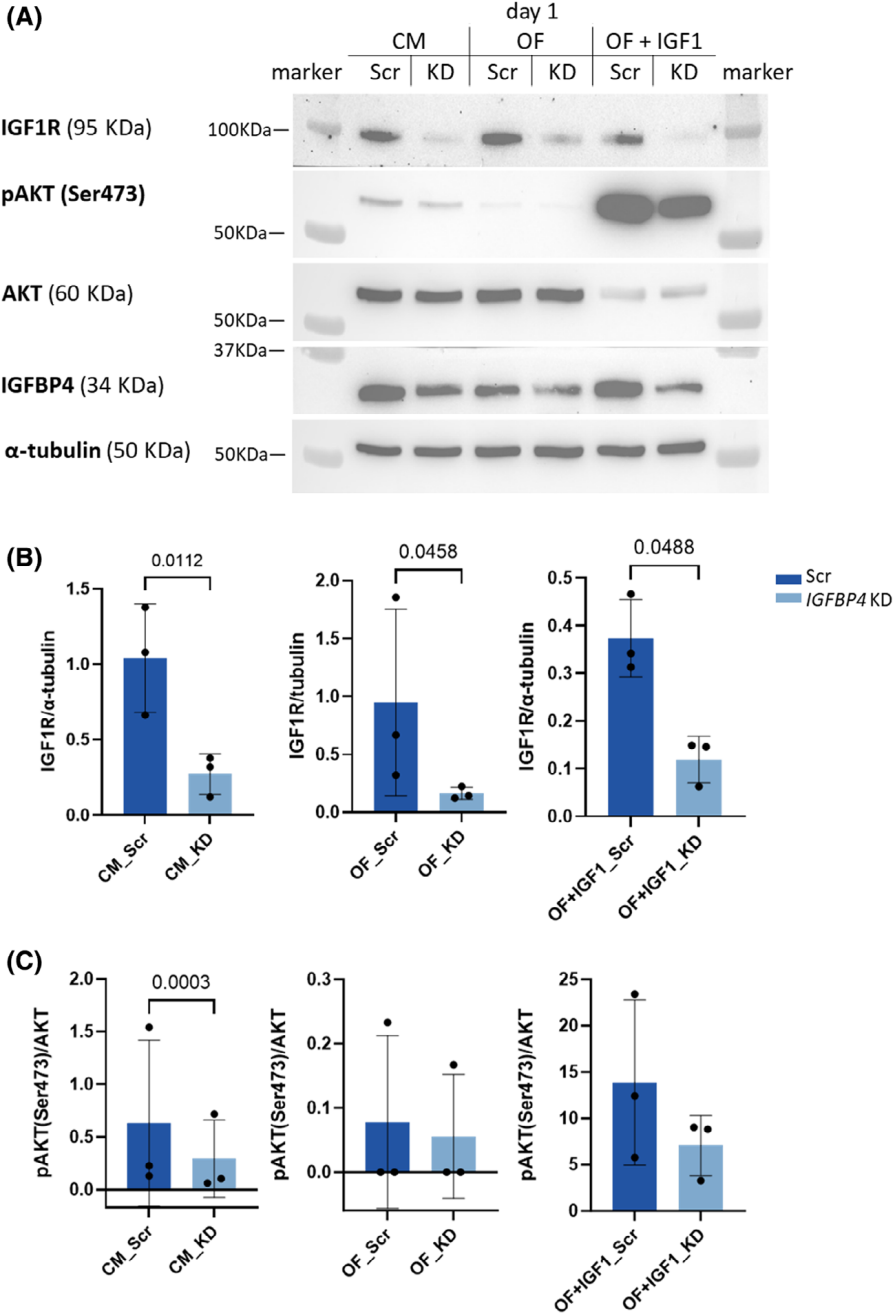
Effect of *IGFBP4* knockdown on AKT pathway in undifferentiated LipPD1 cells. Representative western blot images (A) and densitometric quantification of IGF1R protein (B), phosphorylated AKT (pAKT, Ser473) protein (C) in LipPD1 preadipocytes after *IGFBP4* KD and incubated under culture medium (CM), serum‐free medium (OF), 100 nm IGF1 stimulation (OF + IGF1). IGF1R protein was downregulated under all conditions (*n* = 3, culture medium: *P* = 0.0112, OF medium: *P* = 0.0458, OF+IGF1 medium: *P* = 0.0488, by ratio paired *t*‐test). AKT phosphorylation (Ser473) was downregulated after *IGFBP4* KD under culture medium conditions (*n* = 3, *P* = 0.0003, by ratio paired *t*‐test). Data were presented as mean ± SD.

We next checked if IGF1R/AKT signaling was affected, which could lead to lower proliferation in *IGFBP4* KD cells. Most prominently, we detected lower total IGF1R protein in *IGFBP4* KD cells (*P* < 0.05; Fig. 4A,B), accompanied by slightly lower AKT phosphorylation (*P* < 0.05; Fig. 4A,C). After overnight incubation in serum‐free medium, phosphorylation of AKT was further reduced. IGF1 stimulation induced strong AKT phosphorylation, which was lower in *IGFBP4* KD cells (Fig. 4A,C).

**Fig. 5:**
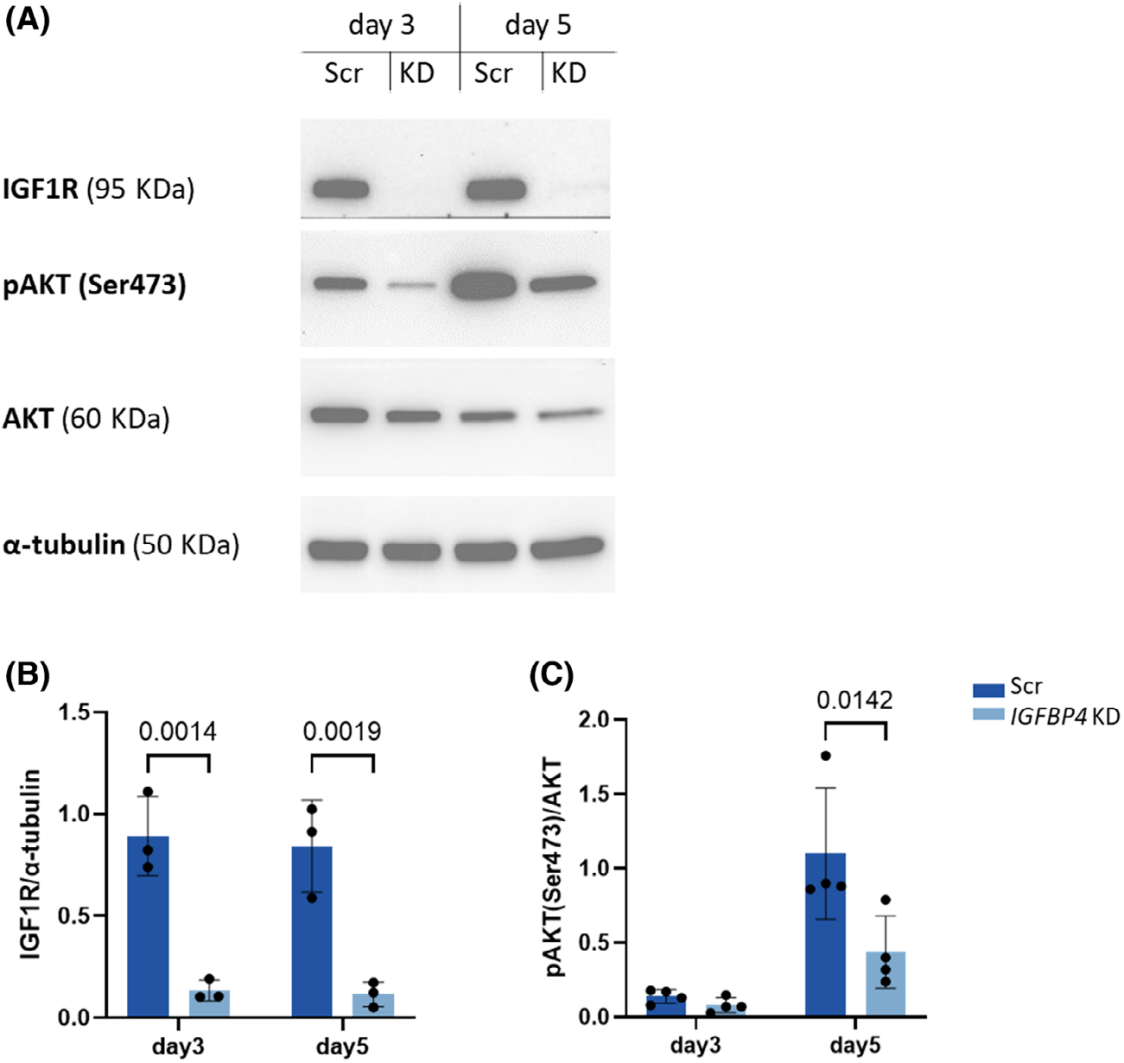
Effect of *IGFBP4* knockdown on AKT pathway in differentiated LipPD1 cells. Representative western blot images (A) and densitometric quantification of IGF1R protein (B); phosphorylated AKT (pAKT, Ser473) protein (C) in LipPD1 adipocytes after *IGFBP4* KD and *in vitro* differentiation for 3 and 5 days. IGF1R protein (B) and pAKT (Ser473) protein (C) expression were downregulated after *IGFBP4* KD. Data were presented as mean ± SD (*n* = 3 for IGF1R, *P* < 0.01, by two‐way ANOVA) (*n* = 4 for pAKT, *P* = 0.0142, by two‐way ANOVA).

During early differentiation, we found similar effects on IGF1R (Fig. 5A,B) and phosphorylated AKT (Fig. 5A,C) that were lower in *IGFBP4* KD cells. Phosphorylated ribosomal protein S6 (Fig. S3) was also decreased in *IGFBP4* KD cells during early adipocyte differentiation compared to control cells.

To exclude cell‐specific effects, we repeated these experiments in two different human adipocellular models: SGBS cells [13] (Fig. S4) and primary human stromal‐vascular fraction (SVF) cells [14] (Fig. S5). *IGFBP4* mRNA level in SGBS cells was reduced by 97% on Day 0 (*n* = 3, *P* = 0.0030) and by 94% on Day 5/6 (*n* = 3, *P* = 0.0163) after *IGFBP4* KD, whereas IGFBP4 protein was downregulated by 27% on average on Day 1. After 6 days, *IGFBP4* KD SGBS exhibited decreased proliferation (6.0 ± 0.8‐fold in KD vs. 13.3 ± 1.8‐fold in controls; *P* = 0.0019). Ki67‐positive SGBS showed a 14% decrease (54.8 ± 5.6% in KD vs. 64.1 ± 9.8% in controls; *P* = 0.0522). In primary SVF cells, on Day 1 (48 h after transfection), *IGFBP4* mRNA was downregulated by 98.9%; whereas IGFBP4 protein showed a minimal decrease. SVF proliferation also declined to 0.77‐fold of controls following *IGFBP4* KD (2.18‐fold in KD vs. 2.84‐fold in controls). Ki67‐positive nuclei showed a slight reduction (66.3% in KD vs. 70.4% in controls).

**Fig. 6:**
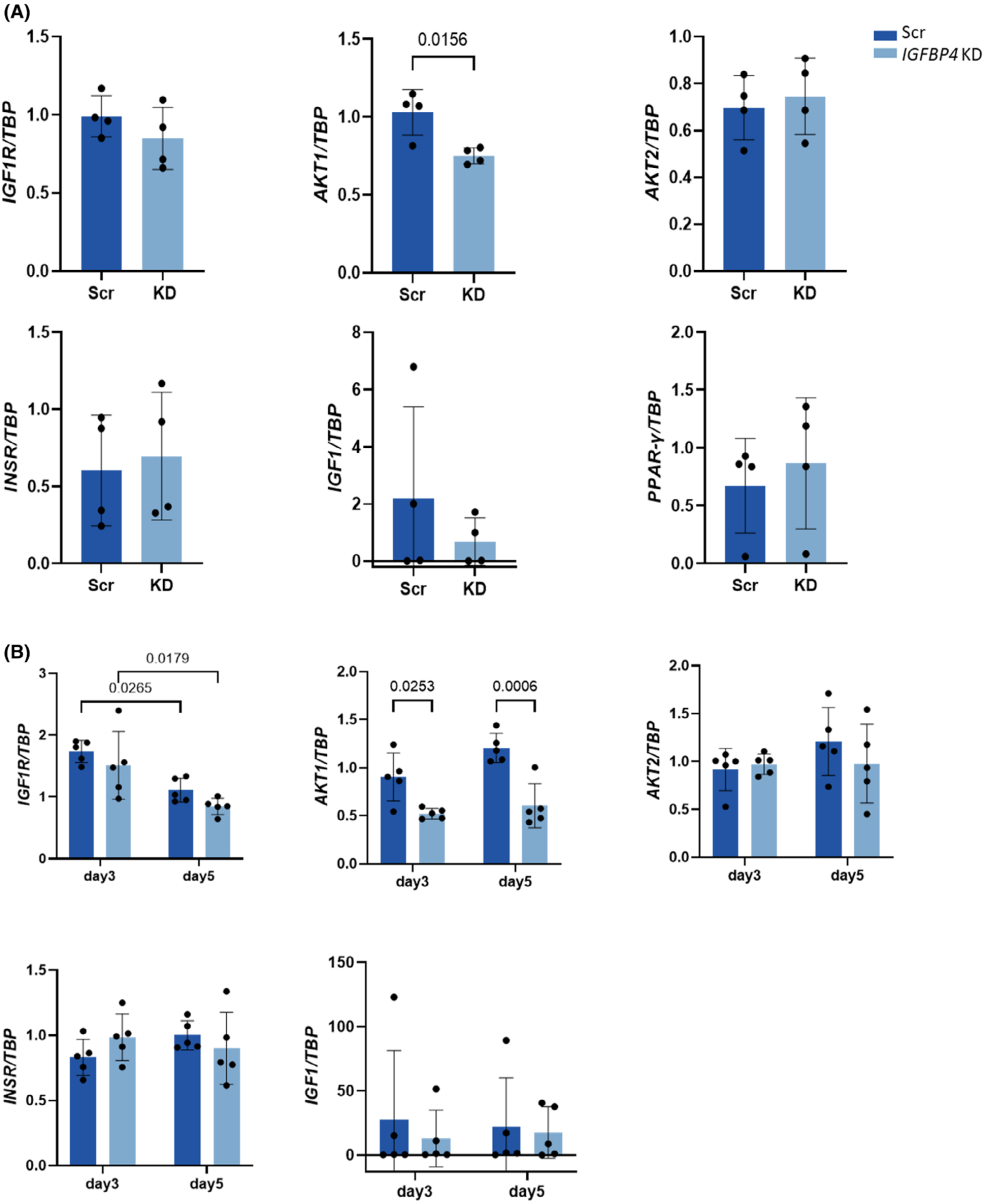
*IGF1R*, *AKT1*, *AKT2*, *InsR*, *IGF1*, *PPAR*γ mRNA levels after *IGFBP4* knockdown. Gene expression from undifferentiated (A) and differentiated (B) LipPD1 cells was measured by qPCR. *AKT1* mRNA, but not *AKT2* mRNA, was downregulated after *IGFBP4* KD. *IGF1R* mRNA was not affected by *IGFBP4* KD. Data were normalized to *TATA‐box binding protein* (*TBP*) and presented as mean ± SD (A: *n* = 4, *P* was defined by paired *t*‐test; B: *n* = 5, *P* < 0.05, by two‐way ANOVA).

To find out whether these differences were due to reduced *IGF1R* and *AKT* mRNA, we checked gene expression. We did not see a difference in *IGF1R, AKT2, INSR*, and *IGF1*, but a decline in *AKT1* expression (*P* < 0.05) after knockdown as compared to control during proliferation (Fig. 6A) and early differentiation (Fig. 6B).

### Adding recombinant IGFBP4 did not rescue IGF1R downregulation after *IGFBP4* KD

Since it was shown previously that IGFBP4 binds and sequesters IGF1 [6, 7, 8], we reasoned that IGF1 will increase after *IGFBP4* KD. We therefore added recombinant human IGF1 to the culture medium to evaluate its effect on IGF1R. Since we noticed that adding recombinant IGF1 to culture medium led to decreased IGF1R protein levels (Fig. S6), we reasoned that increased availability of IGF1 after *IGFBP4* knockdown could lead to less IGF1R protein, and the addition of recombinant IGFBP4 would reverse this effect.

**Fig. 7:**
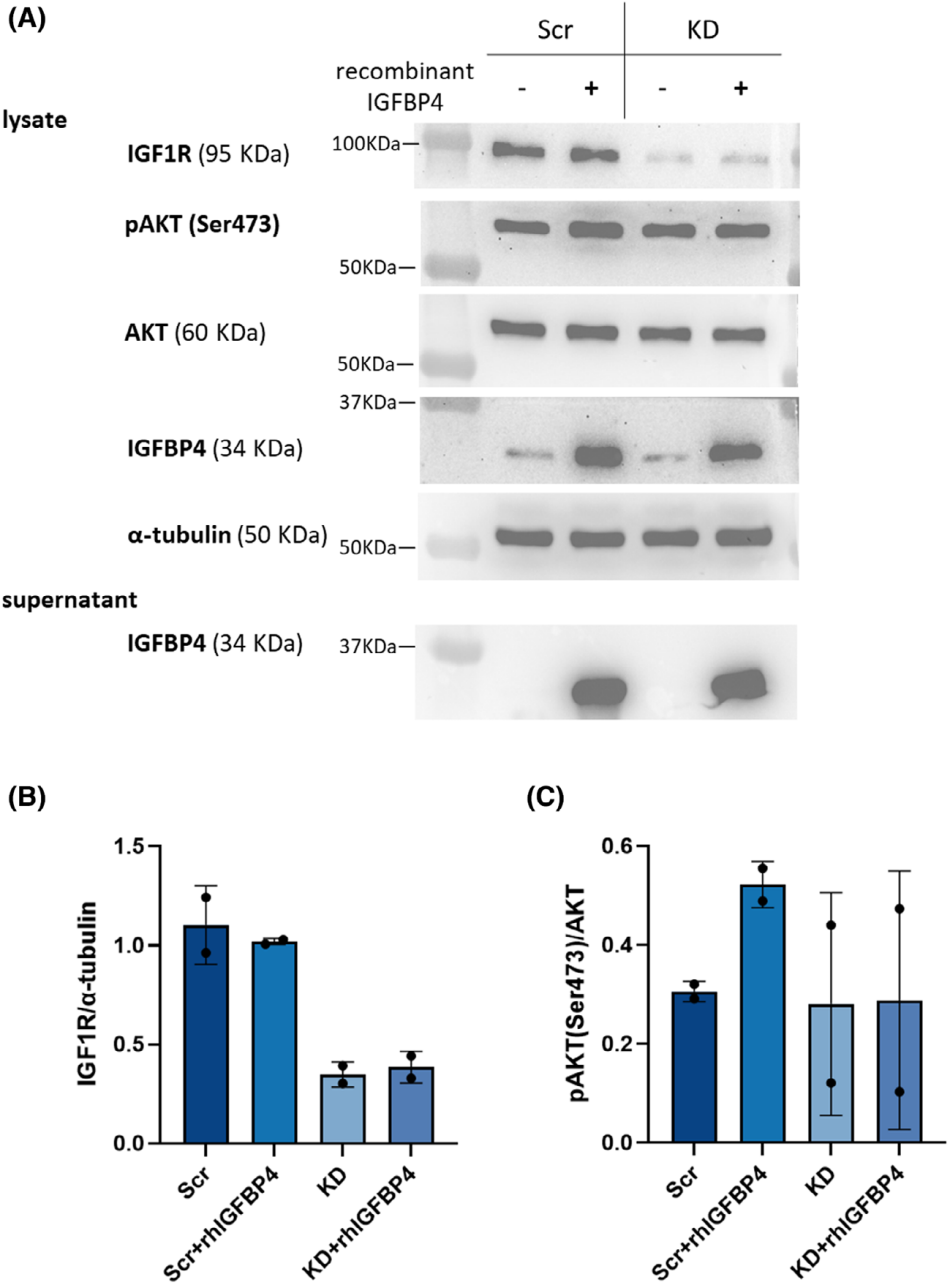
Effect of adding recombinant IGFBP4 on the expression of IGF1R and phosphorylated AKT (pAKT Ser473) in LipPD1 preadipocytes. *IGFBP4* KD and control cells (Scr) were grown in culture medium supplemented with or without recombinant IGFBP4 (1 μg/mL) for 48 h. (A) Representative western blot images of IGF1R, pAKT, AKT, *α*‐tubulin, and IGFBP4 from cell lysates and IGFBP4 in supernatant were shown. (B) Densitometric quantification of IGF1R protein. IGF1R protein was lower after *IGFBP4* KD (Scr vs. KD, Scr + IGFBP4 vs. KD + IGFBP4), whereas supplementation with recombinant IGFBP4 did not significantly alter IGF1R levels in either Scr or KD cells. (Scr vs. Scr + IGFBP4, KD vs. KD + IGFBP4). (C) Densitometric quantification of pAKT (Ser473) protein. Control‐transfected cells with IGFBP4 (Scr + IGFBP4) showed slightly higher pAKT expression. Data were presented as mean ± SD (*n* = 2).

To test this hypothesis, we added recombinant IGFBP4 (1 μg/mL) and observed that IGFBP4 was increased both in lysates and supernatants of cells (Fig. 7A). However, IGF1R (Fig. 7A,B) and phosphorylated AKT (Fig. 7A,C) did not increase in *IGFBP4* KD cells supplemented with recombinant IGFBP4, whereas control‐transfected cells incubated with recombinant IGFBP4 showed a slightly higher phosphorylation of AKT (Fig. 7A,C).

### Inhibition of protein synthesis counteracted IGF1R downregulation after *IGFBP4* KD

**Fig. 8:**
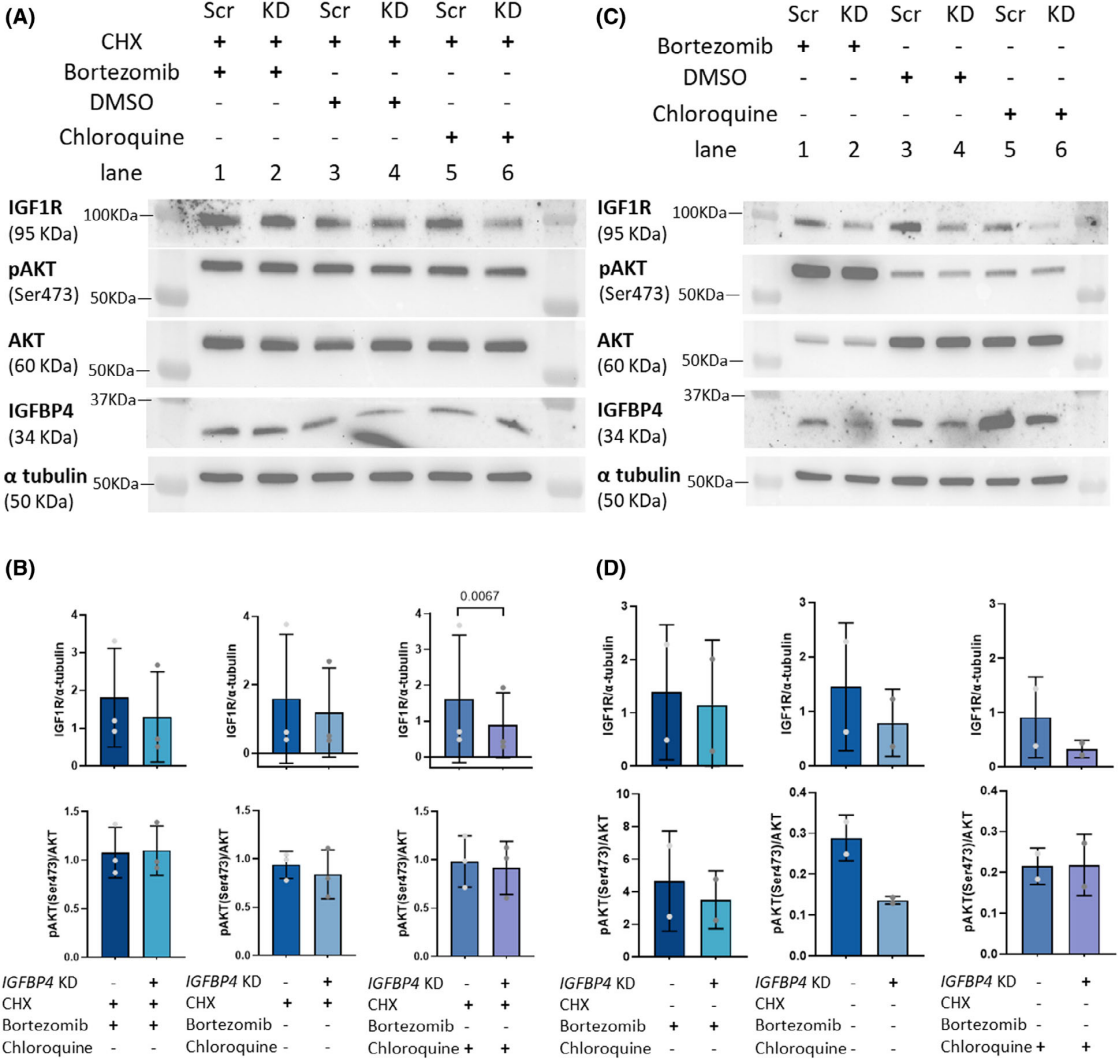
Effect of proteasome and lysosome inhibitors on IGF1R degradation. To inhibit protein *de novo* synthesis, the protein synthesis inhibitor cycloheximide (CHX, 10 μg/ml) was added 30 min before the application of other inhibitors and was maintained for the entire experiment. Then, proteasome inhibitor bortezomib (1 μm, lane 1, 2), solvent control (DMSO, lane 3, 4), and lysosome inhibitor chloroquine (50 μm, lane 5, 6) were added for 6 h. Proteins were extracted and analyzed by western blot. (A) With the application of CHX, IGF1R levels were similar between Scr and KD in the bortezomib‐inhibited and DMSO control groups, while IGF1R level decreased after KD in the chloroquine‐treated group (B, *P =* 0.0067, by ratio paired *t*‐test). AKT phosphorylation remained similar in each condition. (B) Densitometric quantification of IGF1R protein and pAKT (Ser473) protein from (A) was performed using ImageJ and data were presented as mean ± SD (*n* = 3). (C) Without protein synthesis inhibition, IGF1R protein was decreased after *IGFBP4* KD, regardless of the addition of inhibitors. Strong phosphorylation of AKT was shown in the bortezomib‐treated group. (D) Densitometric quantification of IGF1R protein and pAKT (Ser473) protein from (C) was performed using ImageJ and data were presented as mean ± SD (*n* = 2).

We then tested if protein synthesis, proteasomal degradation, or lysosomal degradation were involved in *IGFBP4* KD‐mediated reduction of IGF1R levels. After inhibition of protein *de novo* synthesis by cycloheximide, the previously observed difference between *IGFBP4* KD and control cells was not obvious anymore in the DMSO control and bortezomib‐inhibited groups. In contrast, inhibition of lysosomal protein degradation with chloroquine partly preserved IGF1R downregulation (Fig. 8A). AKT phosphorylation was similar across various conditions (Fig. 8A). As illustrated in the densitometry quantification (Fig. 8B), chloroquine‐treated cells exhibited a decrease in IGF1R levels following the pretreatment of cycloheximide.

Without inhibiting protein synthesis, IGF1R was downregulated following *IGFBP4* KD. AKT phosphorylation was increased, and the decrease of AKT phosphorylation after *IGFBP4* KD cells was inhibited when bortezomib was applied (Fig. 8C). Densitometry quantification of IGF1R and pAKT (Ser473) protein is shown in Fig. 8D.

## Discussion

Our study aimed to explore the impact of *IGFBP4* deficiency during adipogenesis in human preadipocytes. We hypothesized that *IGFBP4* knockdown would promote cell proliferation and differentiation in human preadipocytes by liberating IGF1 to activate IGF1R and the downstream AKT signaling pathway. This premise aligns with IGFBP4's role as a high‐affinity IGF1‐binding protein to sequester IGF1 activities [16].

Contrary to our expectations, *IGFBP4* KD was associated with significantly suppressed preadipocyte proliferation and with reduced IGF1R and phosphorylated AKT levels, which were consistent with decreased cell proliferation. In contrast, we did not observe a significant effect on preadipocyte differentiation. These findings suggest that IGFBP4 may serve as a regulator of human preadipocyte expansion.

The downregulation of IGF1R and phosphorylated AKT may explain the reduced proliferation after *IGFBP4* KD. IGF1R has a role in mediating IGF1 signaling in development, growth, and cancer. Whole‐body *Igfbp4* knockout mice showed growth retardation compared to wild‐type controls [17]. In human studies, children with a pathogenic *IGF1R* variant caused by chromosomal alterations showed growth retardation [18, 19]. On the contrary, a child with three copies of the *IGF1R* gene was born with a 97th percentile birth weight and showed rapid early postnatal growth [19]. Increased *IGF1R* expression is frequently observed in neoplastic growth [20], while anti‐IGF1R antibodies and IGF1R tyrosine kinase inhibitors have been shown to suppress IGF1R signaling and inhibit cancer progression [21].

AKT kinases take part in diverse cellular processes, including cell growth, proliferation, metabolism, survival, glucose uptake, and angiogenesis [22]. The AKT isoforms have overlapping but specific roles. Although AKT1 and AKT2 are both vital for insulin‐stimulated metabolic regulation, the AKT2 isoform‐specific knockdown was shown to reduce proliferation and differentiation in human preadipocytes [23].

As indicated by our live cell imaging readout, IGF1 supplementation did not markedly enhance cell proliferation compared with serum‐free medium but increased cell confluence. This suggests that, despite the substantial reduction in IGF1R level following *IGFBP4* KD, residual IGF1R signaling may still mediate cellular responses, potentially affecting cell adhesion or spreading. A previous study [24] showed that IGF1 stimulation increased integrin expression and enhanced cell adhesion and chemotaxis in hepatocarcinoma cells.

A potential reason for the unchanged differentiation after *IGFBP4* KD could be the decrease of *IGFBP4* KD efficiency over the 9‐day differentiation period. Previous animal research showed that global *Igfbp4* knockout mice exhibited decreased adipogenesis, reduced fat expansion, and blunted IGF1‐mediated AKT phosphorylation [10]. In mouse stromal cells, *Igfbp4* overexpression led to a moderate increase in lipid accumulation, while *Igfbp4* knockdown resulted in a moderate decrease, relative to control differentiated cells [25]. Consistently, expression of adipogenic markers, such as *PPAR‐γ*, *C/EBPα*, *AGPAT2*, and *SREBP1*, was elevated in *IGFBP4* overexpressing cells, but suppressed in *IGFBP4* knockdown cells [25]. Collectively, these findings indicate that IGFBP4 plays a lipogenic role and modulates the expression of adipogenic markers during adipogenesis through the IGF1/AKT pathway. However, this interpretation does not align with our observation that *PPAR‐γ* expression was not significantly influenced in our transient *IGFBP4* KD cell model, nor was lipid accumulation.

Additionally, we observed that *IGFBP4* mRNA expression in LipPD1 preadipocytes exhibited a downward trend during differentiation. However, this is inconsistent with the study by Boney *et al*. [2] who reported that IGFBP3 and IGFBP4 secretion from either mouse primary stromal vascular cells or 3 T3‐L1 cells in culture medium was static and did not change during differentiation or IGF1 stimulation. According to their findings, IGFBP3 and IGFBP4 are not specific to adipogenesis as the modulator for IGF1 [2]. Interspecies variation may underlie the divergent secretory profiles between mouse 3 T3‐L1 cells or primary stromal vascular fraction cells and human preadipocyte models [26].

Besides gradually diminished siRNA efficiency, the distribution of insulin receptor (INSR) and IGF1R could affect the differentiation signaling [27]. During human preadipocyte differentiation, the amount of *INSR* mRNA and total protein increases, while the *IGF1R* gene and total protein remain at an approximately constant level [27]. IGF1R signaling might, therefore, lose its dominance in regulating differentiation events, regardless of the downregulation of IGF1R protein after *IGFBP4* KD.

Since IGF1R protein was downregulated after *IGFBP4* KD, we investigated potential reasons and tested different strategies to reverse this process. We assumed that IGF1 was liberated after *IGFBP4* KD. To mimic IGF1 release and to test its effect on IGF1R, we applied recombinant IGF1 to the culture medium. In contrast to the decrease in IGF1R and AKT phosphorylation observed after *IGFBP4* knockdown, recombinant IGF1 induced AKT phosphorylation, although IGF1R levels were also downregulated. This demonstrates that free IGF1 is associated with lower IGF1R, regardless of whether IGF1 was liberated by *IGFBP4* KD or from direct supplementation. Moreover, exogenously added IGF1 maintained AKT activation, suggesting that *IGFBP4* KD disrupts postinternalization receptor trafficking. These findings align with Romanelli *et al*.'s work [28], in which they suggested that IGF1R internalization induced by a ligand is required for AKT phosphorylation, and the following IGF1R recycling sustains AKT phosphorylation in glial progenitors. Under the condition of *IGFBP4* KD, IGF1R recycling might be interrupted, which caused prolonged IGF1R loss. IGF1R downregulation explains blunted AKT signaling despite IGF1 availability, as increased IGF1 would normally enhance IGF1R/AKT pathway activation.

Another possible mechanism could be related to ligand concentration‐dependent IGF1R internalization routes. For the receptor tyrosine kinase epidermal growth factor receptor (EGFR), a low concentration of EGF (1.5 ng/mL) induces EGFR internalization by the clathrin‐dependent route without ubiquitination and sustains the activation of downstream AKT and ERK signaling. Higher concentrations of EGF (100 ng/mL), conversely, cause EGFR internalization by a clathrin‐independent pathway leading to receptor ubiquitination and degradation [29]. By analogy, we suppose that exogenously added IGF1 triggers clathrin‐dependent IGF1R internalization (analogous to low‐dose EGFR dynamics), while an increase of IGF1 after *IGFBP4* knockdown may lead to IGF1R ubiquitination and degradation without sustainable AKT activation.

We aimed to restore IGF1R levels with recombinant IGFBP4, considering that the affinity of IGFBP4 to IGF1 is higher than that between IGF1 and IGF1R [30]. Contrary to our expectation, recombinant IGFBP4 failed to rescue IGF1R in *IGFBP4* knockdown cells. Conversely, in control‐transfected cells, recombinant IGFBP4 slightly boosted phosphorylated AKT without depleting IGF1R.

There are several potential mechanisms underlying the failure of recombinant IGFBP4 rescue. First and foremost is the bioactivity of recombinant IGFBP4. Since IGFBP4 binds to IGF1 mostly through its N‐terminal region and contains an N‐linked glycosylation site [16], variations in glycosylation, protein conformation, and stability may directly influence IGF‐binding activity. We used recombinant IGFBP4 from both insect cells and mammalian cells and observed the same inability to rescue IGF1R protein expression. Therefore, differences in expression system or post‐translational modification alone are unlikely to fully explain the failure of rescue.

Additionally, there exists the possibility of the inhibitory function of recombinant IGFBP4 being counteracted by pregnancy‐associated plasma protein‐A (PAPP‐A) that cleaves recombinant IGFBP4 to release IGF1 [31]. How PAPP‐A activity influences the IGFBP4‐IGF1 system in our adipocyte models and whether supplementation with protease‐resistant IGFBP4 would elicit different effects will be subjects for further studies.

Another noteworthy result was that *IGF1R* mRNA expression was not influenced by *IGFBP4* knockdown, indicating that a post‐translational modification of IGF1R protein might underlie the inconsistency between gene and protein expressions. This prompted us to investigate the potential involvement of IGF1R protein degradation.

Protein synthesis and degradation are dynamically coupled processes. Referring to the study by Knutson *et al*., cycloheximide did not affect INSR internalization or recycling but interfered with its degradation [32]. Considering the structural similarities of INSR and IGF1R and hybrid receptors consisting of half of each receptor, we might argue that cycloheximide may interfere with IGF1R degradation while preserving IGF1R recycling, maintaining downstream AKT phosphorylation. In our cell model, suppressing protein synthesis diminished the downregulation of IGF1R protein after *IGFBP4* KD. This supports the assumption that protein synthesis is vital for protein degradation.

IGF1R degradation exhibits cell‐type‐dependent specificity, which was revealed by blocking proteasome or lysosome functions in a study by Carelli *et al*. They reported that IGF1R degradation occurs via the ubiquitin‐proteasome pathway in a neoplastic human lung cell line, whereas non‐neoplastic human cells utilize a lysosome‐mediated route [33]. We used LipPD1 cells that were isolated from lipoma tissues of a patient with phosphatase and tensin homolog hamartoma tumor syndrome (PHTS) [12]. Nonetheless, lipomas are considered a benign overgrowth, and our results are in line with the results obtained by Carelli *et al*. for non‐neoplastic cells [33].

Dai *et al*. reported that protein degradation is regulated by protein synthesis [34]. They showed that inhibition of protein synthesis by cycloheximide induced phosphorylation of AKT and its downstream E3 ubiquitin ligase MDM2, consequently promoting the downstream protein degradation via the ubiquitin‐proteasome pathway [34]. Their findings align with our observation that suppression of protein synthesis activates AKT. However, phosphorylated AKT was less degraded when proteasomal activity was inhibited by bortezomib, with this effect being more pronounced in the absence of cycloheximide.

For the IGF1R, both proteasomal and lysosomal degradation pathways have been described [35]. In our study, neither bortezomib nor chloroquine alone was sufficient to prevent IGF1R degradation following *IGFBP4* KD. In contrast, pretreatment with cycloheximide alone abolished *IGFBP4* KD‐mediated IGF1R downregulation, while combining protein synthesis and proteasomal inhibition did not have an additional effect. This is contrary to the previously reported ubiquitin‐proteasome‐mediated degradation of IGF1R [35]. How chloroquine facilitates the degradation of IGF1R under protein synthesis suppression conditions is a subject for further studies.

Having described how *IGFBP4* KD affects IGF1R/AKT signaling, it is necessary to mention that IGFBP4 functions through both IGF1‐dependent and ‐independent ways [36]. An *in vivo* study proposed that systemic administration of IGFBP4 stimulates bone formation by shifting IGF from the 150 KDa complex of IGF1/IGFBP3/Acid‐labile subunit (ALS) to the 50 KDa IGF1/IGFBP4 complex, which makes it possible to cross the vascular endothelial barriers to targeted tissues [37]. Localized IGF1 is then released upon subsequent proteolysis of IGFBP4 [38, 39]. If we assume the same process taking place in adipose tissue, *IGFBP4* KD might result in less complex accumulation, therefore decreasing downstream AKT signaling and adipogenesis.

Beyond IGF1 ligand binding, IGFBP4 was shown to exert IGF1‐independent effects through canonical Wnt/β‐catenin signaling [11], which intersects with the AKT pathway at key nodes [40]. Until now, the presence of an IGFBP4 receptor has not been shown, nor has IGFBP4 binding to components of the extracellular matrix as IGFBP5 does [36]. Therefore, the involvement of a potential IGF1‐independent mechanism explaining the effects of *IGFBP4* KD remains to be shown by further studies.

One of the limitations in our experiments is the use of siRNA to knock down *IGFBP4* transiently. As the downregulation of IGFBP4 protein was not maintained during the entire duration of *in vitro* adipocyte differentiation, permanent gene silencing techniques should be considered in future studies to test the effect of IGFBP4 loss on this process. Potential changes of cell survival or cell death cannot be excluded, as they were not directly assessed in the current study.

## Conclusion

Collectively, our data support a model where IGF1—irrespective of whether it was liberated by *IGFBP4* KD or supplied exogenously—induces IGF1R downregulation. We speculate that IGF1R protein expression may depend on the presence of IGFBP4 and IGF1 levels. When IGFBP4 maintains IGF1 at a proper level, IGF1R trafficking permits sustained AKT signaling, whereas *IGFBP4* KD is associated with attenuated IGF1R signaling. Our findings point to IGFBP4 as a novel therapeutic target for mitogenic and metabolic disorders associated with IGF1R dysfunction. Further studies are needed to elucidate the recycling mechanism of IGF1R, and how IGFBP4 is involved in the processes relating to IGF1R internalization, trafficking, and AKT phosphorylation in preadipocytes. Our results suggest an involvement of newly synthesized proteins and the lysosomal degradation pathway in IGF1R downregulation.

## Conflict of interest

All authors declare no conflicts of interest.

## Author contributions

YJG and AG were involved in writing – original draft preparation. YJG, SR, AK, and DLD were involved in experimental work. YJG, CB, and AG were involved in visualization. AG, WK, and DLD were involved in supervision. All authors were involved in draft review.

## Supporting information


**Fig. S1.** LipPD1 cells proliferation after 6 days in serum‐free medium +/− IGF1.
**Fig. S2.** *IGFBP4* gene expression and IGFBP4 protein during differentiation in LipPD1 cells.
**Fig. S3.** Western blot for phosphorylated ribosomal protein S6 and ribosomal S6 protein.
**Fig. S4.** SGBS cell proliferation and IGF1R pathway after *IGFBP4* KD.
**Fig. S5.** Stromal‐vascular fraction (SVF) cell proliferation and IGF1R pathway after *IGFBP4* KD.
**Fig. S6.** Effect of adding recombinant IGF1 on IGF1R and phosphorylated AKT (pAKT, Ser473).

## Data Availability

The data that support the findings of this study are available in the Figures, Tables, and the Supporting Information.
