# Supplementary material for: Effects of IGFBP4 deficiency on human preadipocyte proliferation and differentiation through the IGF1R/AKT pathway
Source: FEBS Open Bio. 2026 Jun 8:10.1002/2211-5463.70282. Online ahead of print. doi: 10.1002/2211-5463.70282 (PMC13399132; doi:10.1002/2211-5463.70282)
Supplement: Supplementary file 1 — Fig. S1. LipPD1 cells proliferation after 6 days in serum‐free medium +/− IGF1. Fig. S2. IGFBP4 gene expression and IGFBP4 protein during differentiation in LipPD1 cells. Fig. S3. Western blot for phosphorylated ribosomal protein S6 and ribosomal S6 protein. Fig. S4. SGBS cell proliferation and IGF1R pathway after IGFBP4 KD. Fig. S5. Stromal‐vascular fraction (SVF) cell proliferation and IGF1R pathway after IGFBP4 KD. Fig. S6. Effect of adding recombinant IGF1 on IGF1R and phosphorylated AKT (pAKT, Ser473). [file FEB4-9999-0-s001.docx]

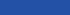

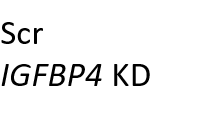

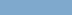


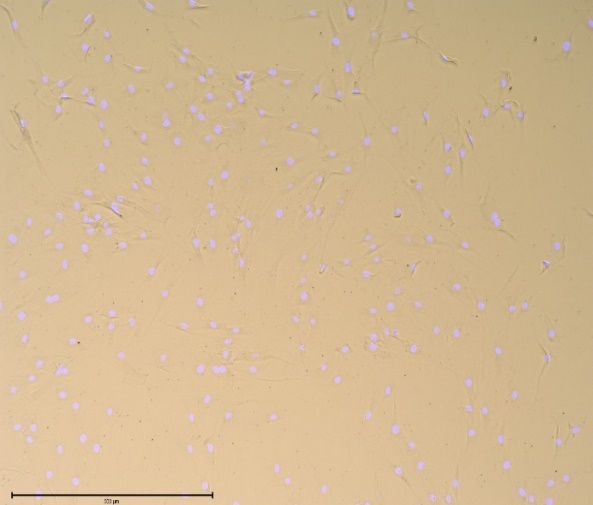

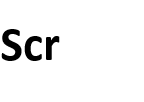


**Supplementary Figure 1: LipPD1 cells proliferation after 6 days in serum-free medium +/- IGF1**

(A) LipPD1 cells were grown under different conditions — OF: serum-free medium; OF+IGF1: plus 100 nM IGF1 — for 6 days, fixed, stained with Hoechst and counted by Image J. Data were normalized to the mean of each Scr or KD group from day 0 and presented as mean ± SD (n = 5).
(B) LipPD1 cell counting 24h after *IGFBP4* KD. LipPD1 cells were fixed, stained with Hoechst staining on the day after *IGFBP4* KD (day 0) and counted by Image J. Data were presented as mean ± SD (n = 8).
(C) Presence of senescence-activated β-galactosidase was assessed in Scr and *IGFBP4* KD cells as well as positive control cells (*TSC1* knockout fibroblasts; blue arrows indicate senescent cells) (n = 4, Blue: cell nuclei, scale bar = 500 µm).


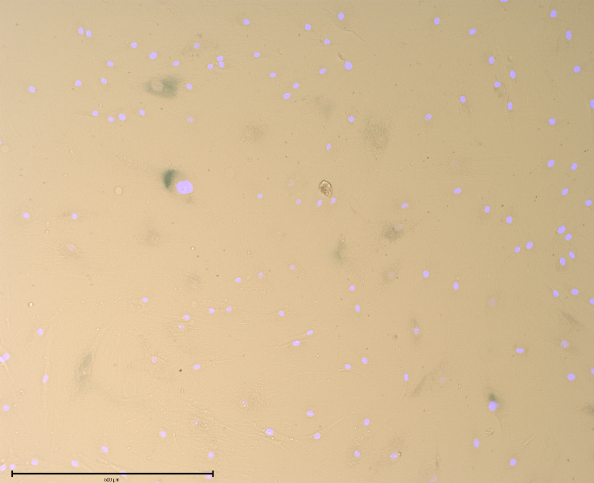


***TSC1* Knockout on fibroblasts**


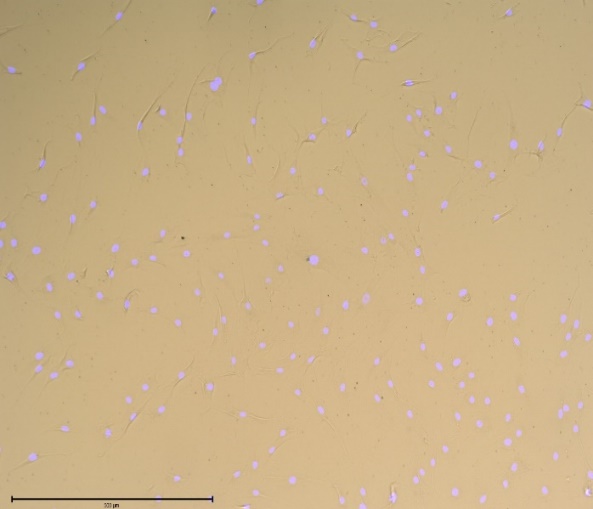


***IGFBP4* KD**

**(C)**

**(B)**

**(A)**

**Supplementary Figure 2: *IGFBP4* gene expression and IGFBP4 protein during differentiation in LipPD1 cells**

(A) *IGFBP4* mRNA levels from differentiated LipPD1 cells were compared to those from undifferentiated cells. *IGFBP4* mRNA level in LipPD1 cells assessed by quantitative PCR (qPCR) on day 3, day 8 and day 12 during differentiation. During differentiation, *IGFBP4* mRNA was the highest on day 3, and then went downward. *IGFBP4* mRNA from differentiated cells was higher than that from undifferentiated cells on day 3 (n = 3 for day 3, n = 2 for day 8, n = 1 for day 12). Data were presented as mean ± SD.
(B) *IGFBP4* knockdown efficiency on mRNA level assessed by qPCR (n = 2, by one-way ANOVA) and Western blot images for IGFBP4 and IGF1R protein after *IGFBP4* KD on day 3 and day 9 of differentiation. IGFBP4 protein expression decreased after *IGFBP4* KD. The difference in IGFBP4 protein between *IGFBP4* KD and Scr controls on day 9 appeared to be smaller than that on day 3. Data were presented as mean ± SD (n=1).

**(A)**


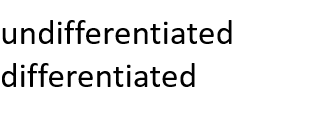


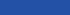

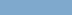


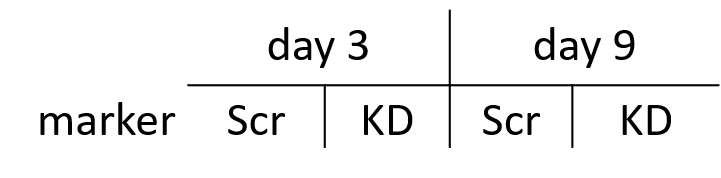


**(B)**


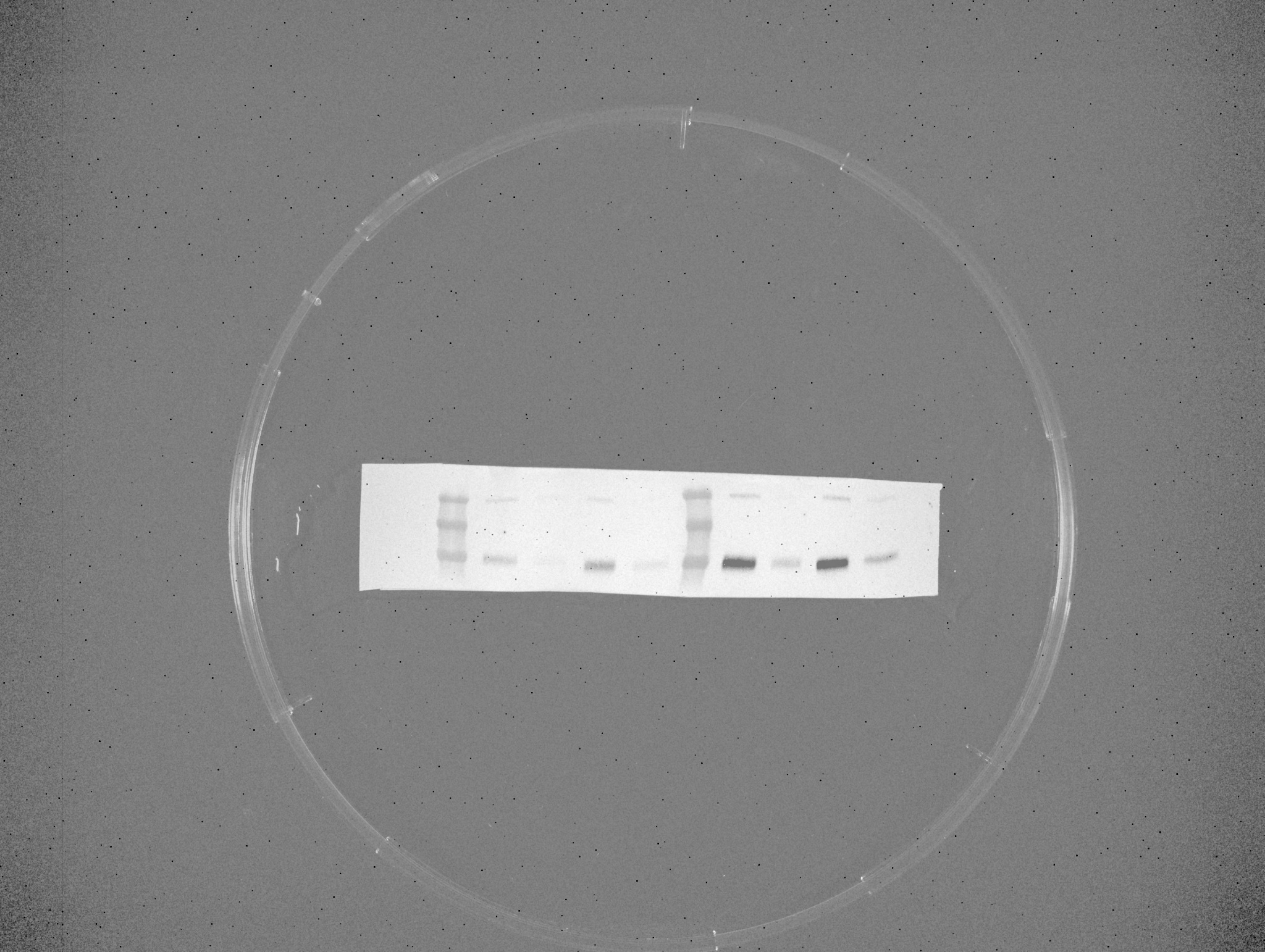


**IGF1R**(95 KDa)

100KDa—


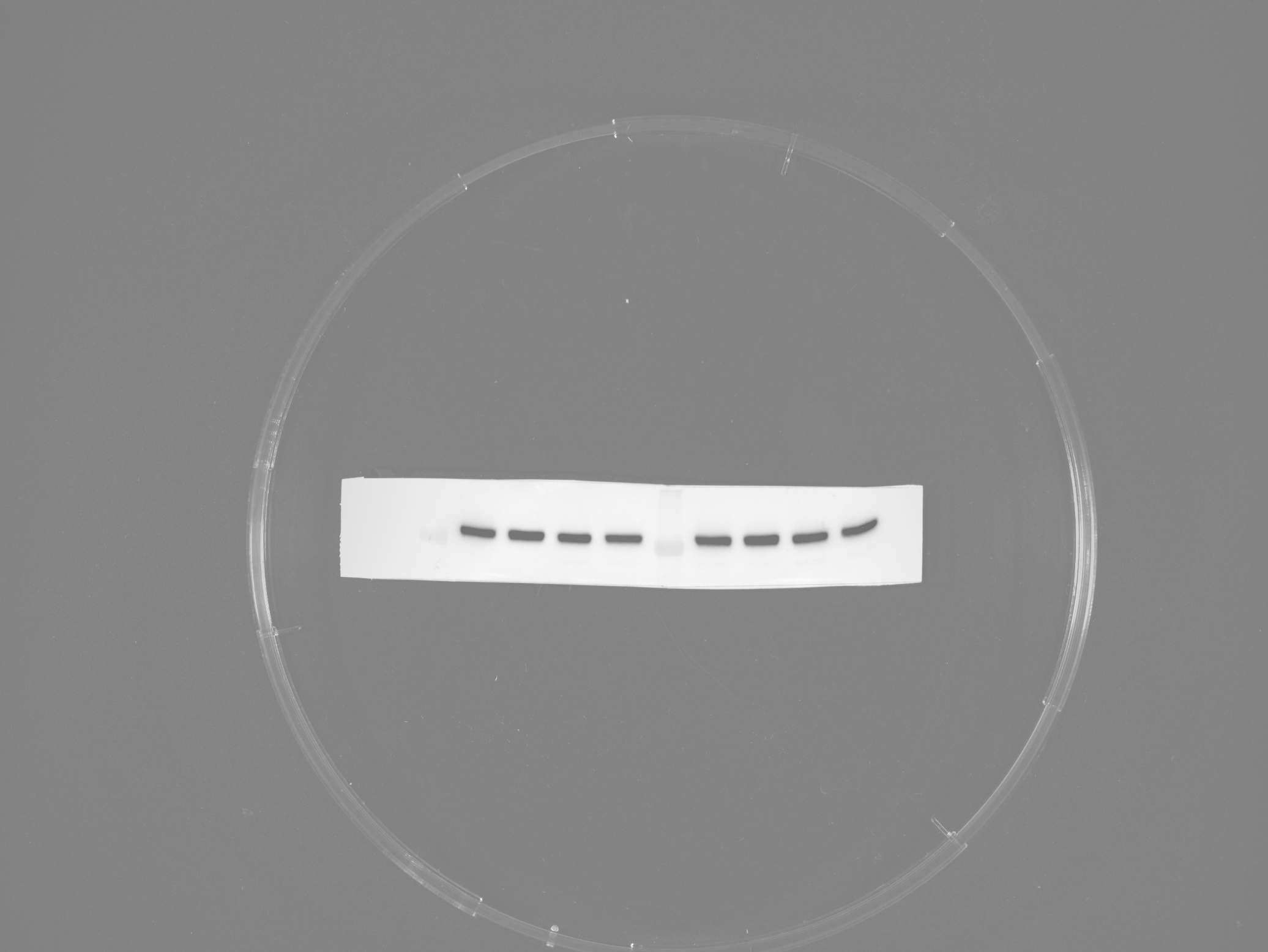


50KDa—

**α-tubulin**(50 KDa)


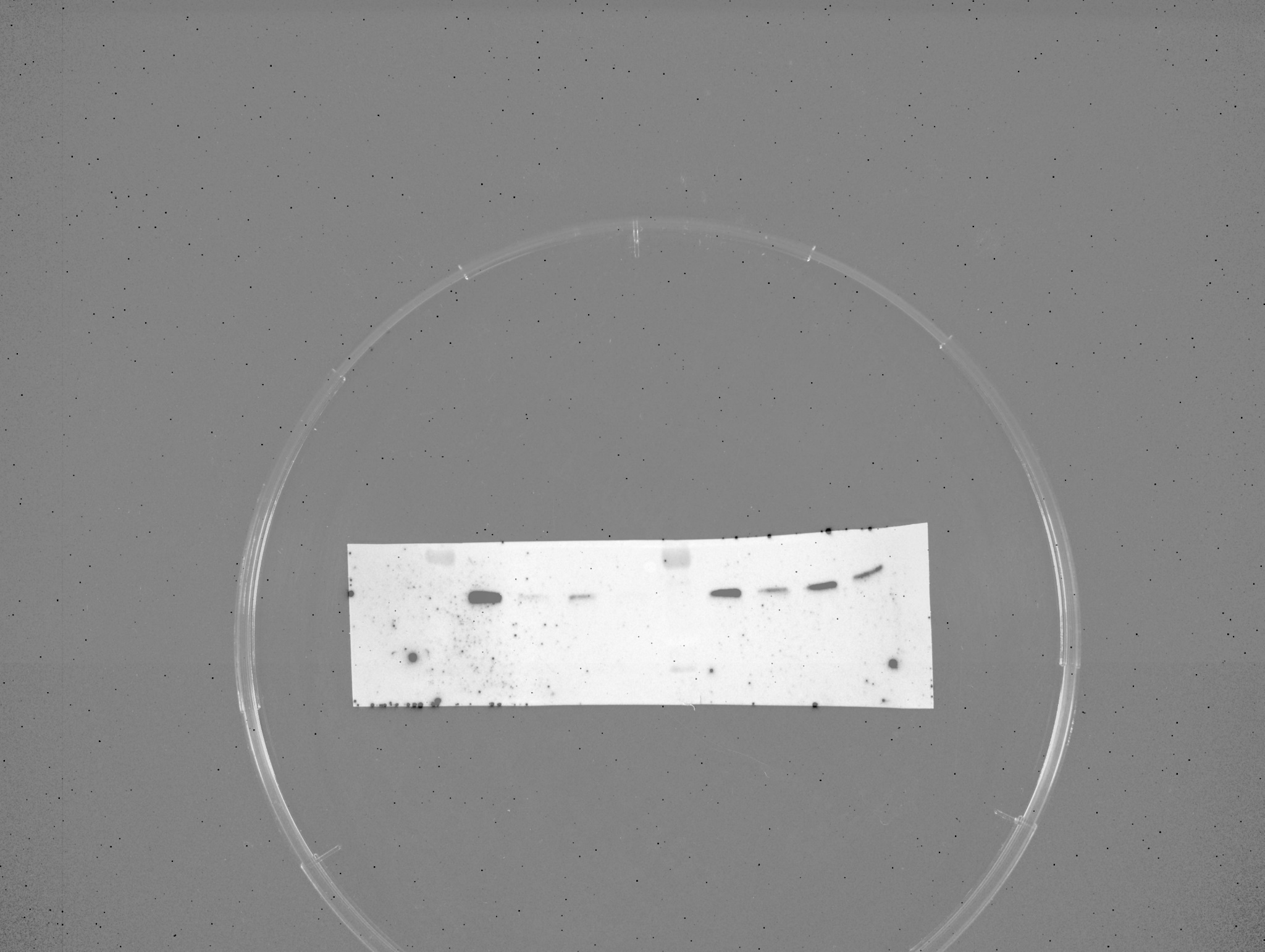


37KDa—

**IGFBP4**(34 KDa)

**Supplementary Figure 3: Western blot for phosphorylated ribosomal protein S6 and ribosomal S6 protein.**

Western blot images (A) and analysis of phosphorylated S6 (pS6) protein (B) and S6 protein (C). pS6 expression was slightly decreased after *IGFBP4* KD during differentiation. One representative experiment out of three independent experiments was shown. Data were presented as mean ± SD (n = 3).

**(A)**


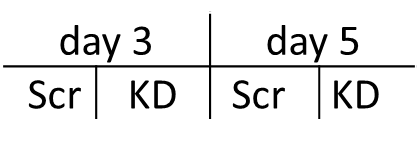


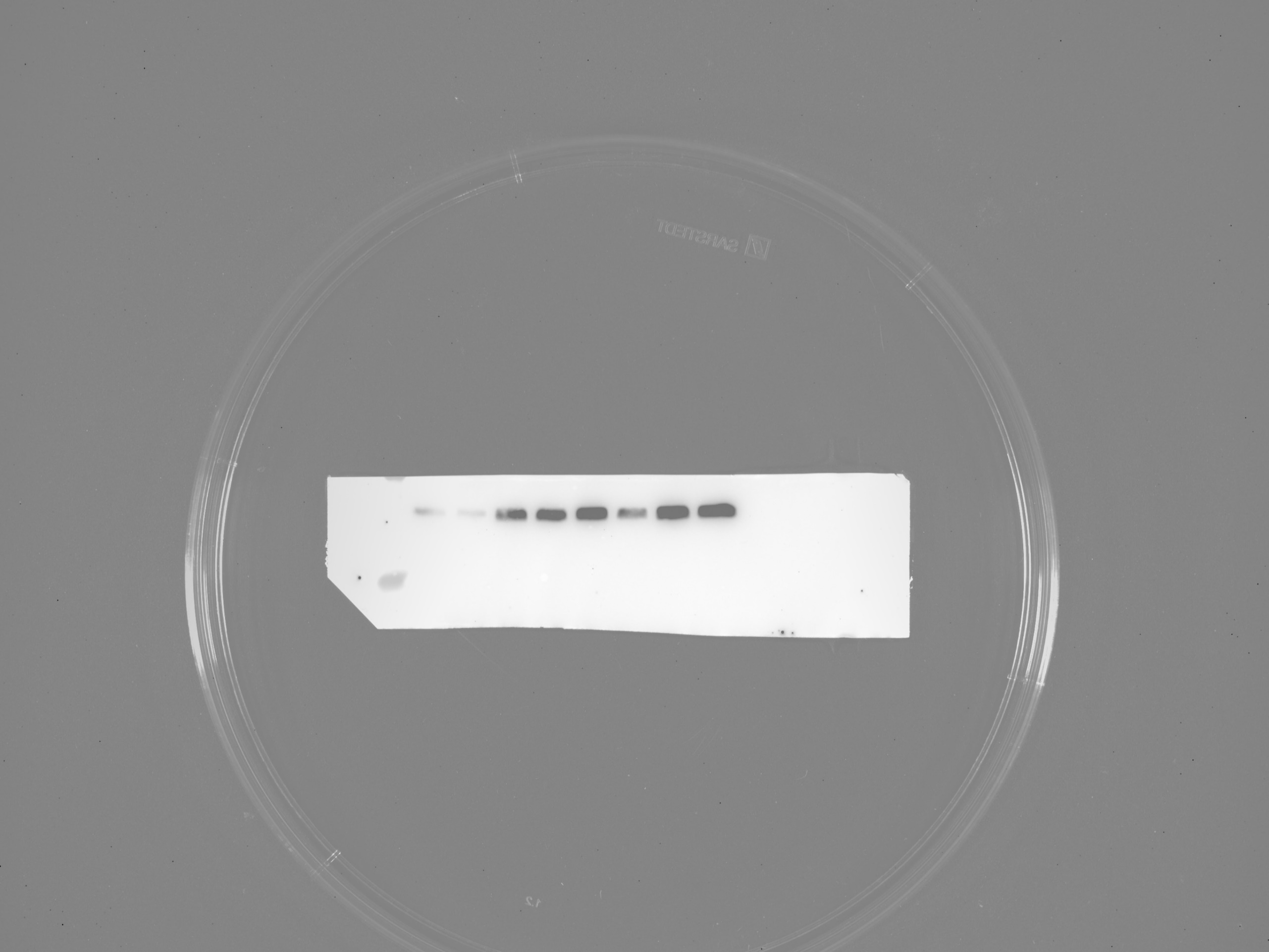


**pS6**


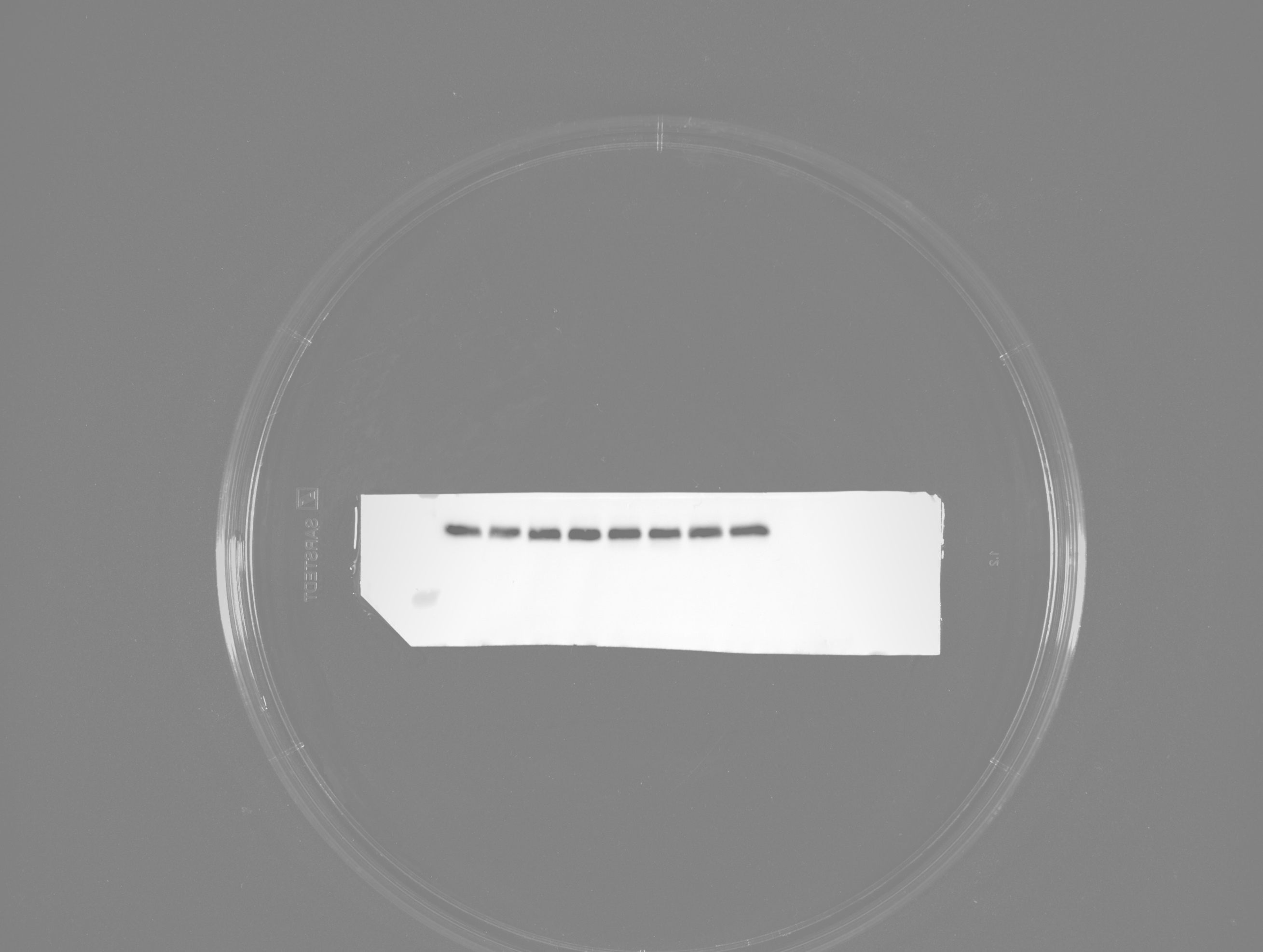


**S6 (32 KDa)**


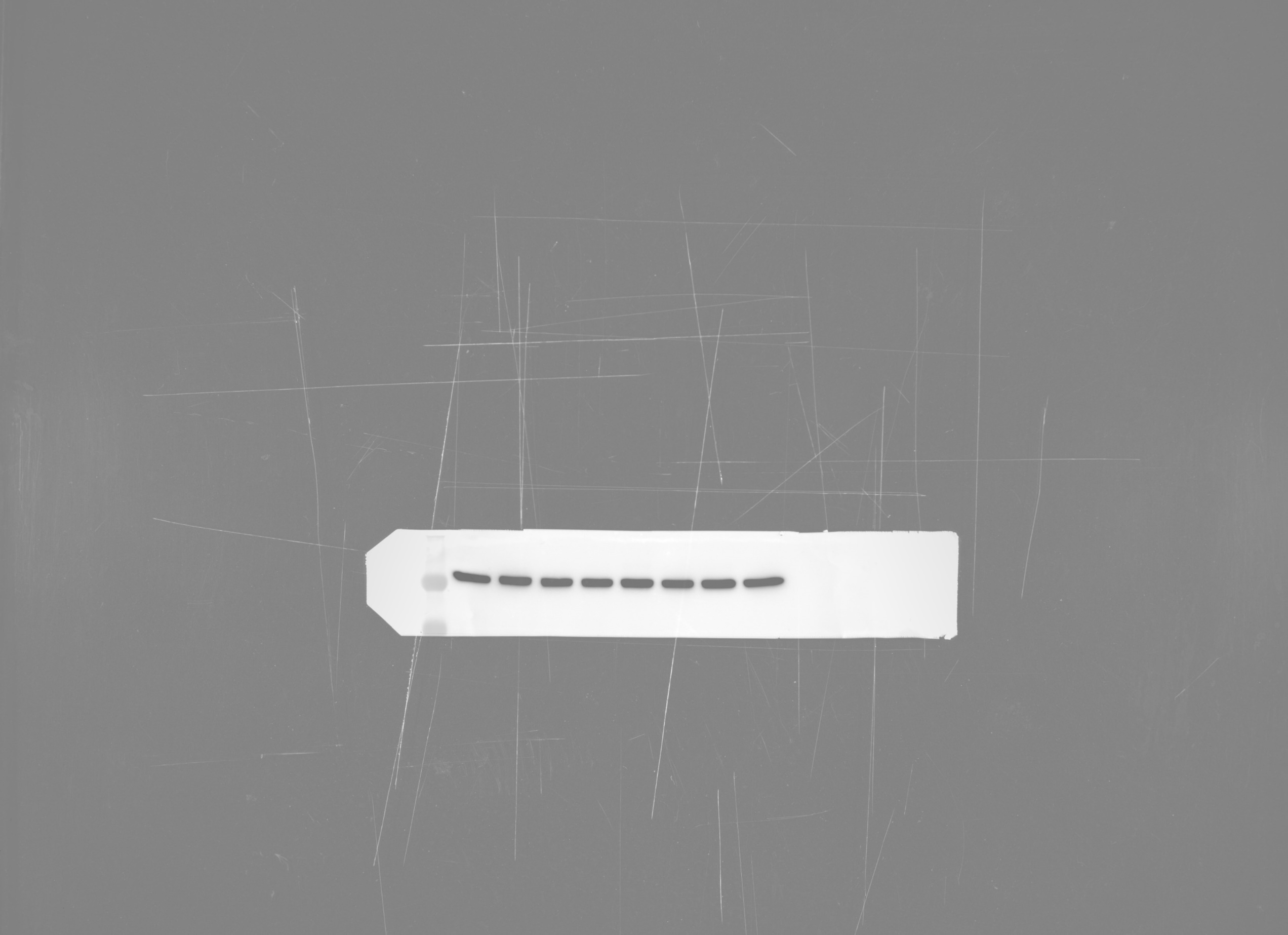


**α-tubulin (50 KDa)**

**(C)**

**(B)**


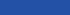

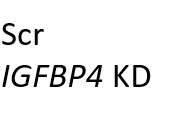

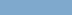


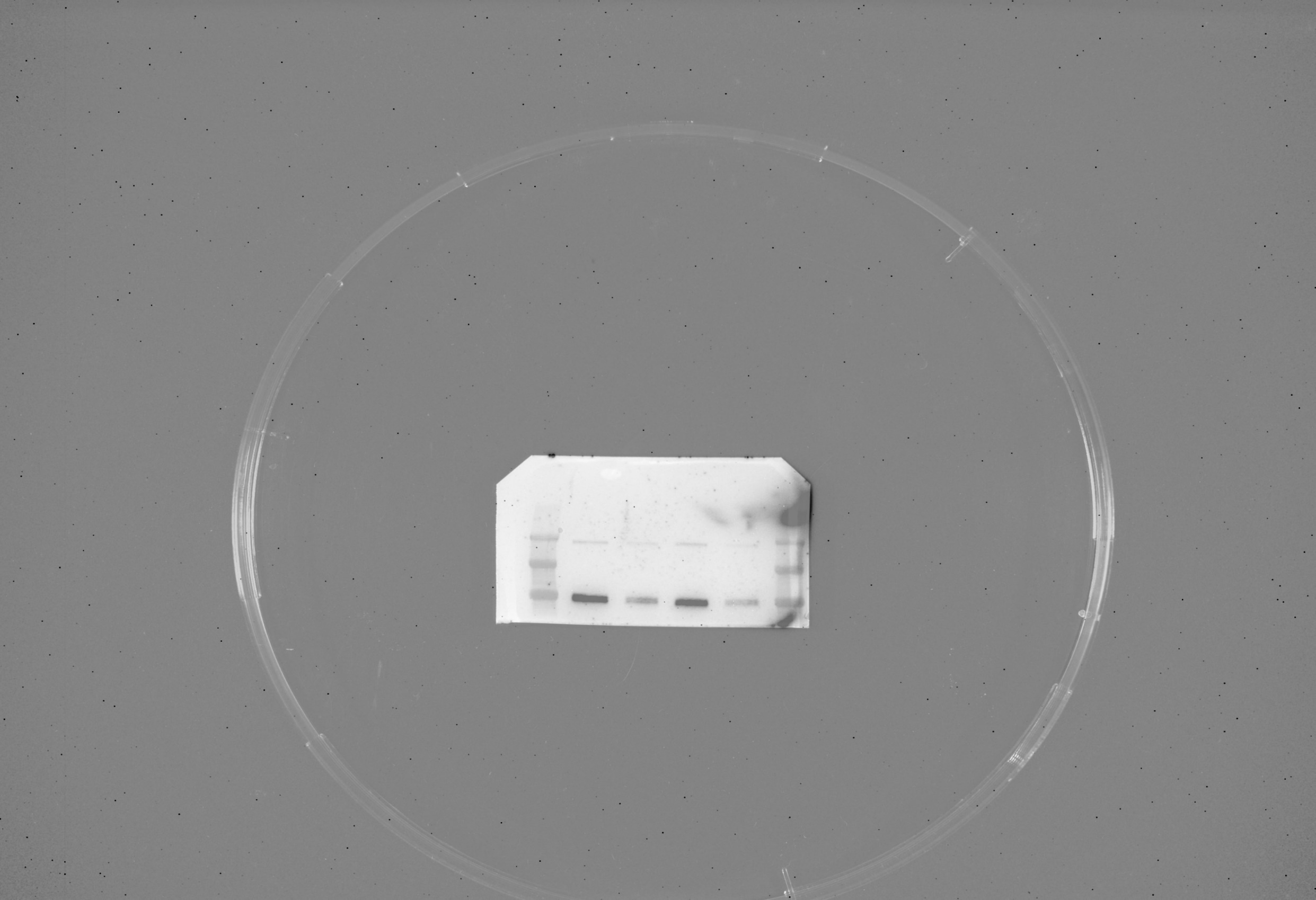

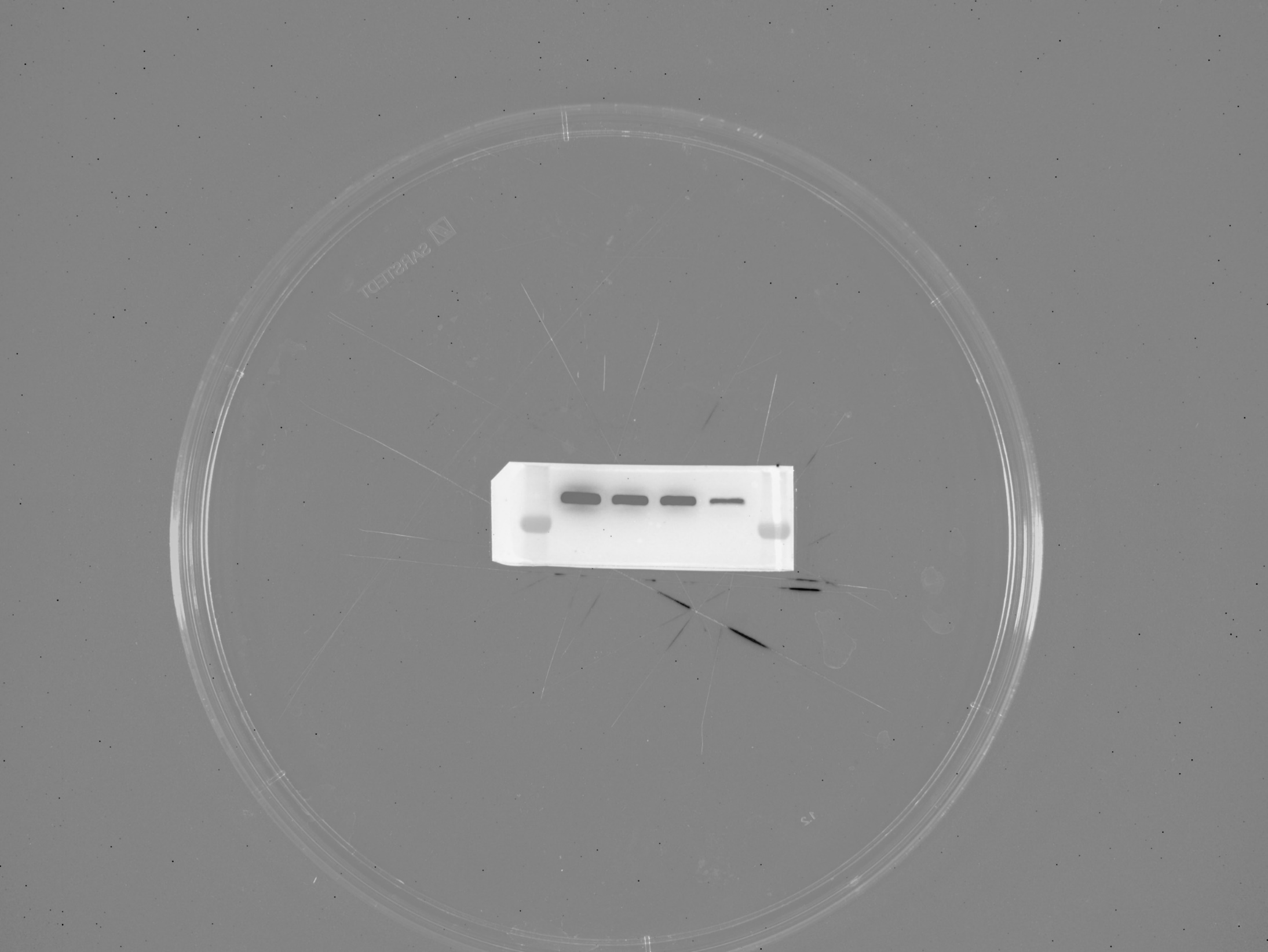

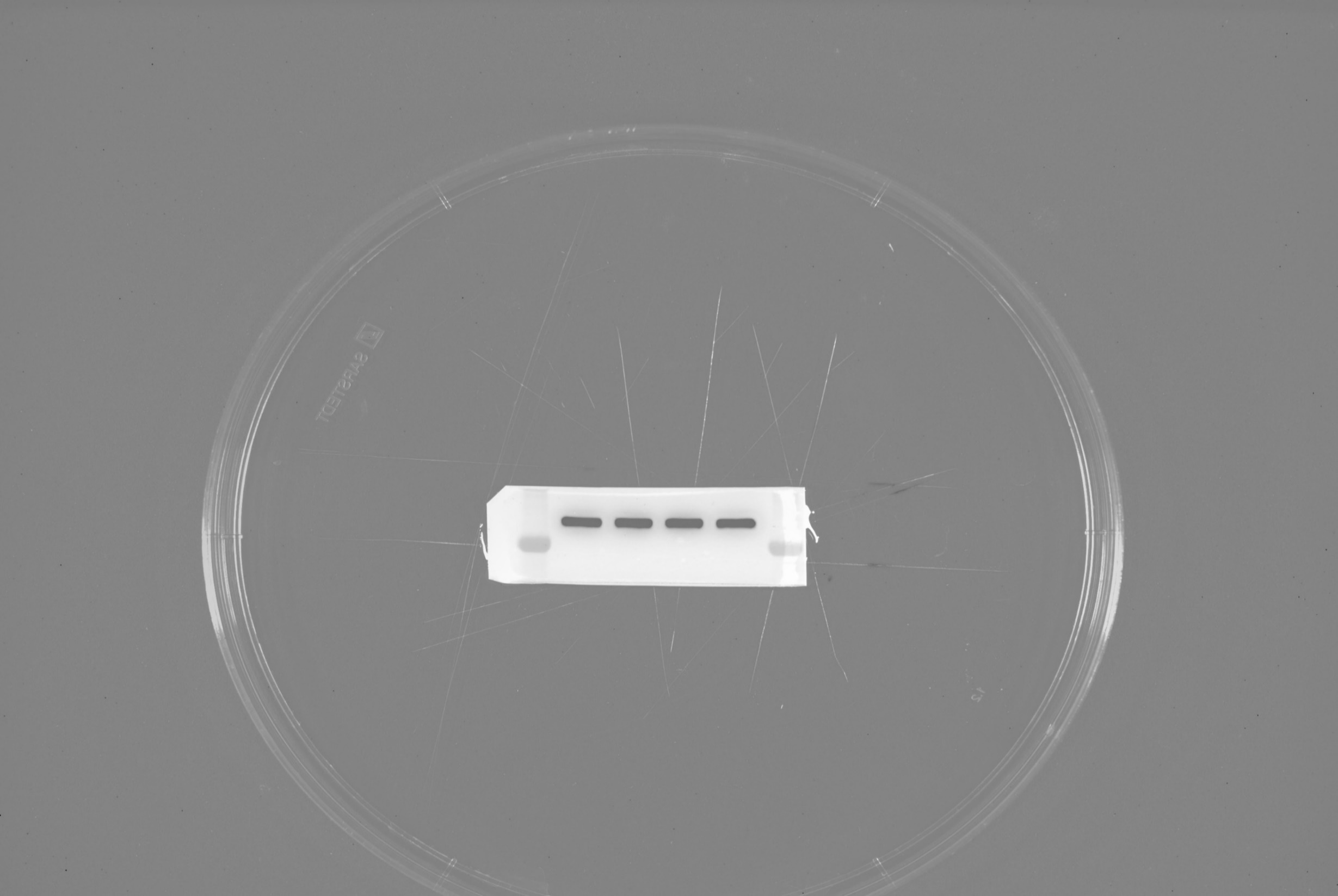

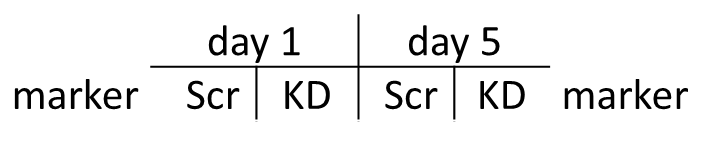

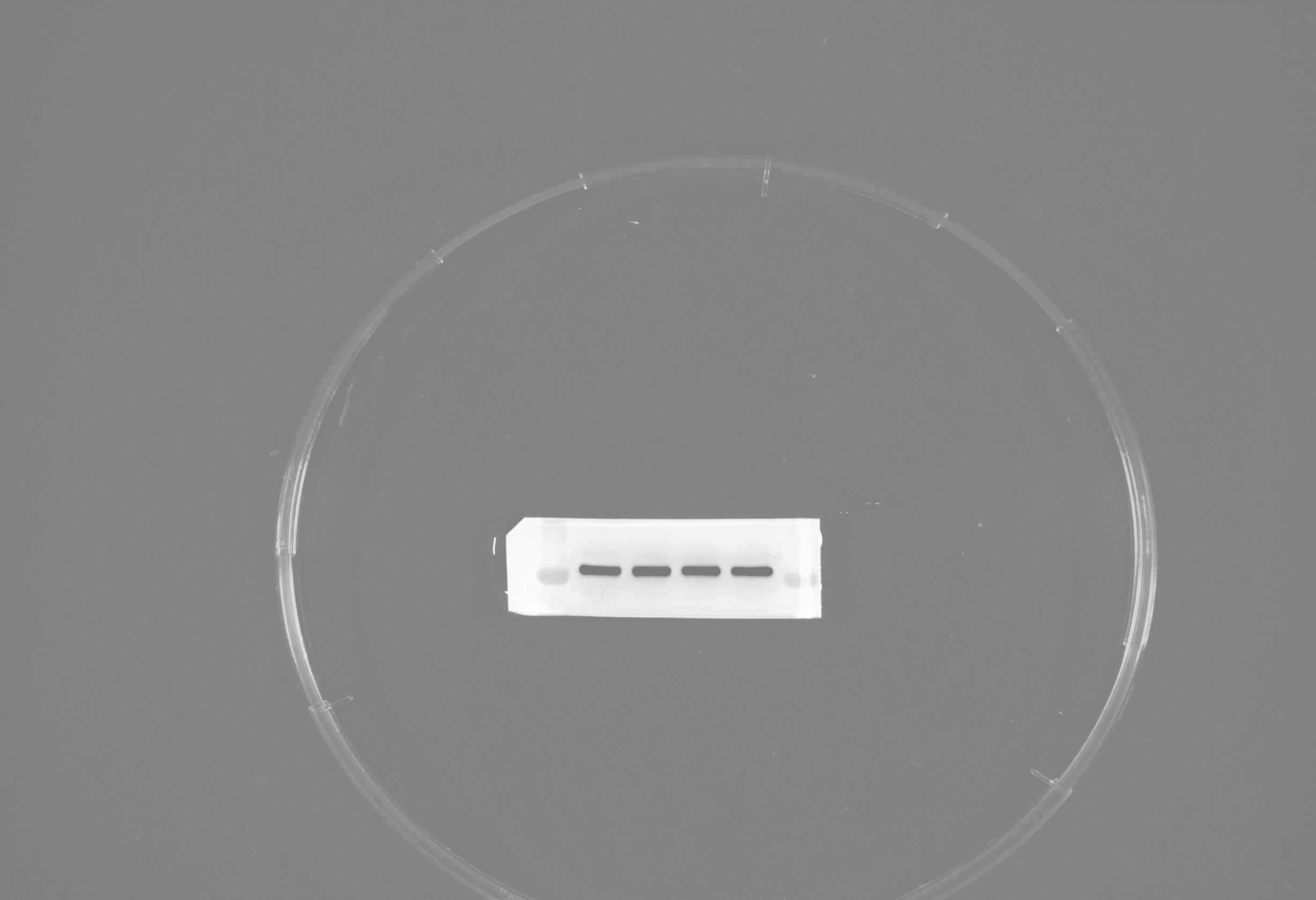




50KDa—

50KDa—

50KDa—

100KDa—

**(A)**

**(D)**

**Supplementary Figure 4: SGBS cell proliferation and IGF1R pathway after *IGFBP4* KD**

(A) Representative western blot images from SGBS cells are shown. IGF1R and pAKT (Ser473) protein decreased after *IGFBP4* KD (n=3).
(B) *IGFBP4* KD efficiency on mRNA level in SGBS cells was assessed by quantitative PCR (qPCR) on day 0 (24h after transfection) and day 5 or day 6. *IGFBP4* mRNA level in SGBS cells was reduced after *IGFBP4* KD (n = 3, by 97% on day 0, *p* = 0.0030 and by 94% on day 5/6, *p* = 0.0163). Data were normalized to the average of *TATA-box binding protein* (*TBP*) and *hypoxanthine phosphoribosyltransferase* (*HPRT*). Significant differences were determined by ratio paired *t*-test.
(C) Cell proliferation was assessed by cell counting and Ki67 immunofluorescence staining. After 6 days, *IGFBP4* KD SGBS cells exhibited decreased proliferation (6.0 ± 0.8-fold in KD vs. 13.3 ± 1.8-fold in controls; *p* = 0.0019, by ratio paired *t*-test). Cell proliferative index was determined as the percentage of Ki67-positive cells and it was decreased by 14% after *IGFBP4* KD (54.8 ± 5.6 % in KD vs. 64.1 ± 9.8 % in controls; *p* = 0.0522, by ratio paired *t*-test). Data were presented as mean ± SD (n = 3).
(D) IGF1R pathway components were quantified by densitometry on day 1 (48h after transfection) and day 5/6. IGFBP4 protein was downregulated by 27% on average on day 1. IGF1R protein was downregulated on day 0 and day 5/6 (n = 3, on day 1: *p* = 0.0224, by one-way ANOVA). AKT phosphorylation (Ser473) was downregulated (n = 3, *p* = 0.0367 on day 1, by one-way ANOVA) and during proliferation (n = 3, *p* = 0.0105, by one-way ANOVA). Data were presented as mean ± SD.

**(B)**

**(C)**

**α-tubulin (50 KDa)**

**IGFBP4 (34 KDa)**

**AKT (60 KDa)**

**pAKT (Ser473)**

**IGF1R (95 KDa)**

37KDa—


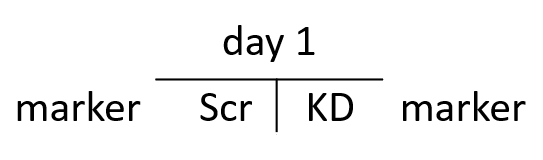


**Supplementary Figure 5: Stromal-vascular fraction (SVF) cell proliferation and IGF1R pathway after *IGFBP4* KD**

(A) Western blot images from SVF cells are shown. IGF1R and pAKT (Ser473) protein slightly decreased after *IGFBP4* KD (n=1).
(B) *IGFBP4* KD efficiency on mRNA level in SVF cells was assessed by quantitative PCR (qPCR) on day 0 (24h after transfection). *IGFBP4* mRNA was downregulated by 98.9 % (n=1).
(C) Cell proliferation was assessed by cell counting and Ki67 immunofluorescence staining. After 6 days, SVF proliferation declined to 0.77-fold of controls following *IGFBP4* KD (2.18-fold in KD vs. 2.84-fold in controls). Cell proliferative index was determined as the percentage of Ki67-positive cells and showed a slight reduction (66.3 % in KD vs. 70.4 % in controls).
(D) IGF1R pathway components were quantified by densitometry on day 1 (48h after transfection). While IGFBP4 protein showed a minimal decrease, IGF1R and pAKT (Ser473) declined after *IGFBP4* KD (n=1).

**(A)**


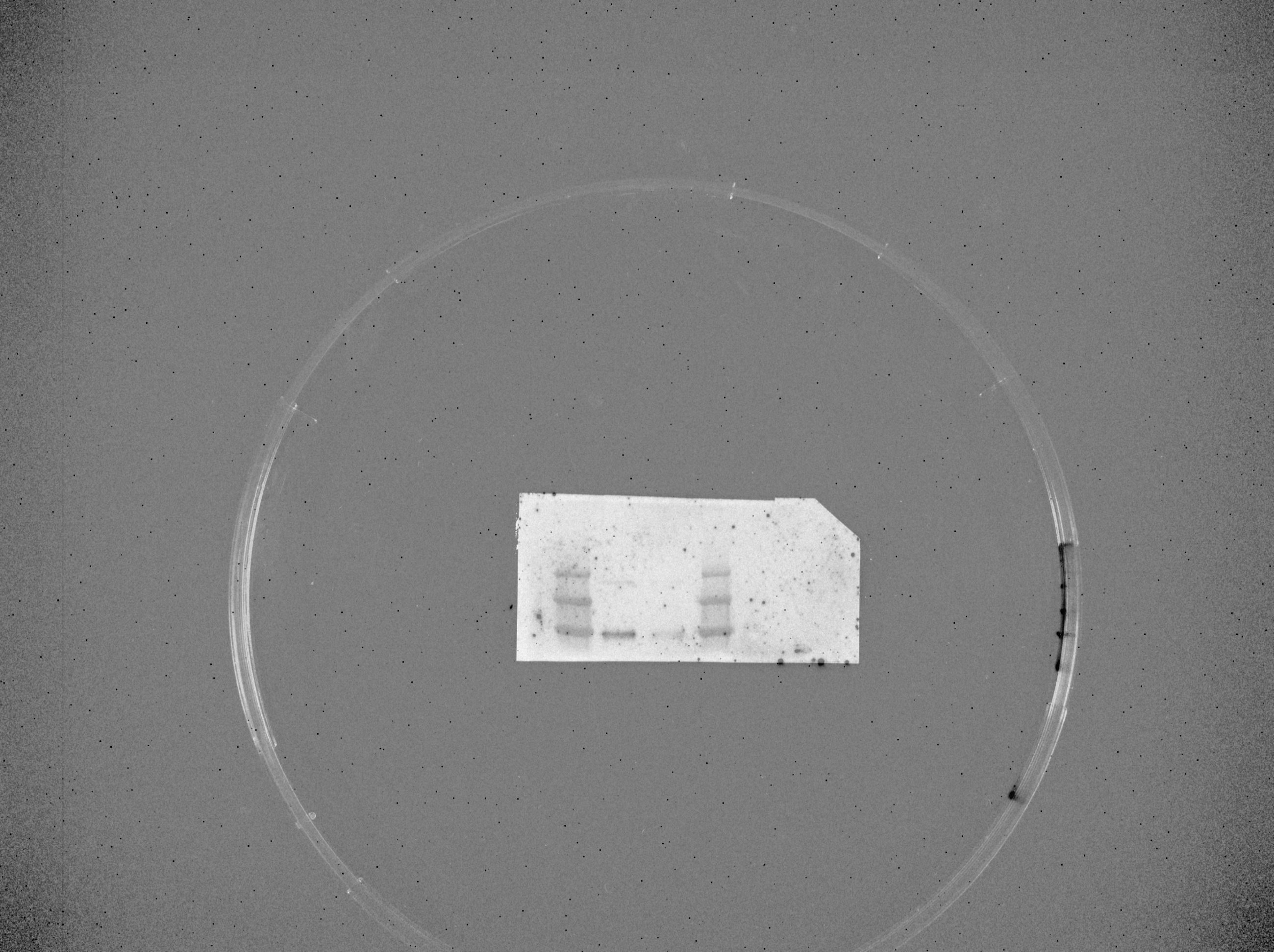


100KDa—

**IGF1R (95 KDa)**


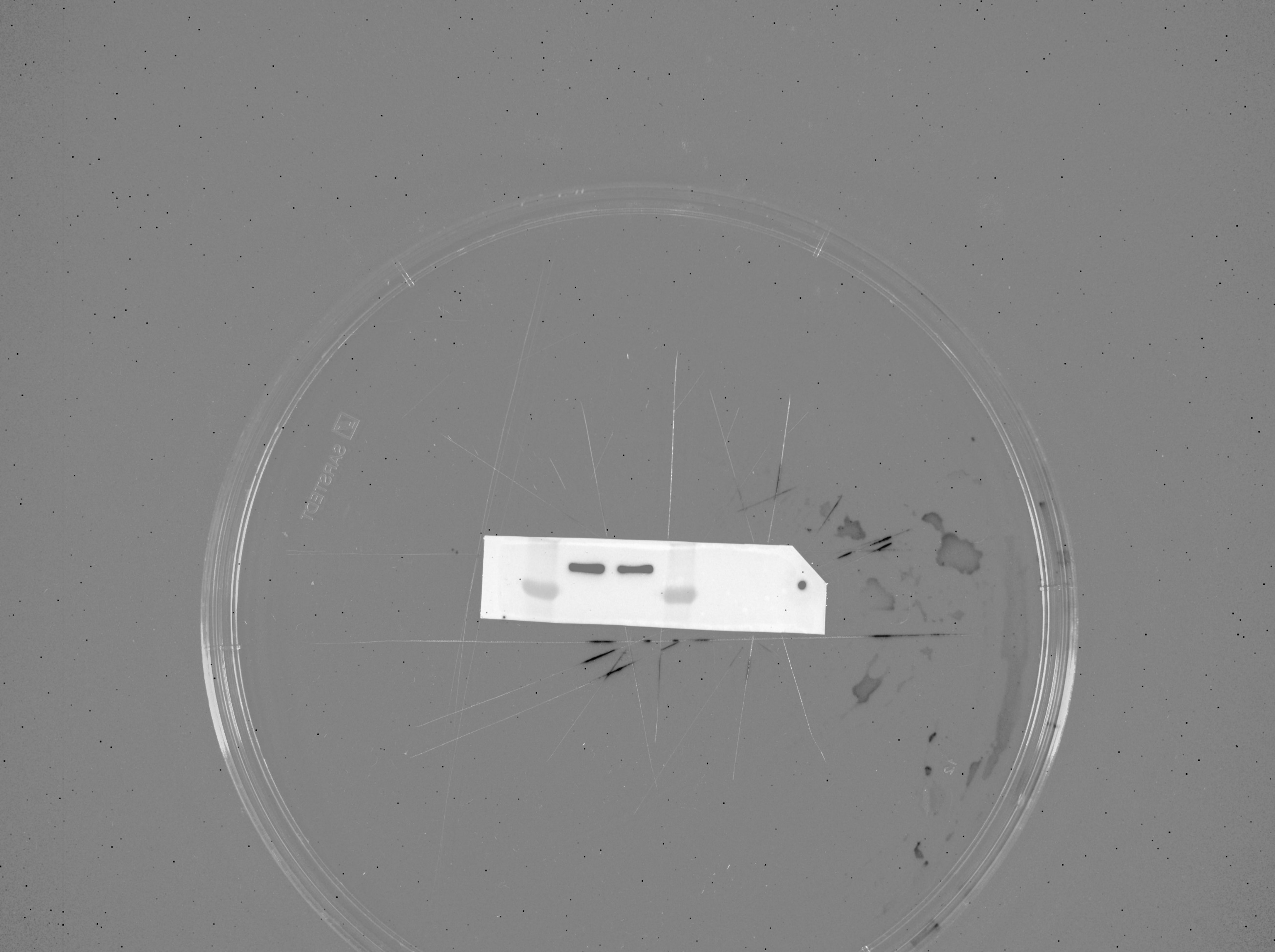


**pAKT (Ser473)**

50KDa—


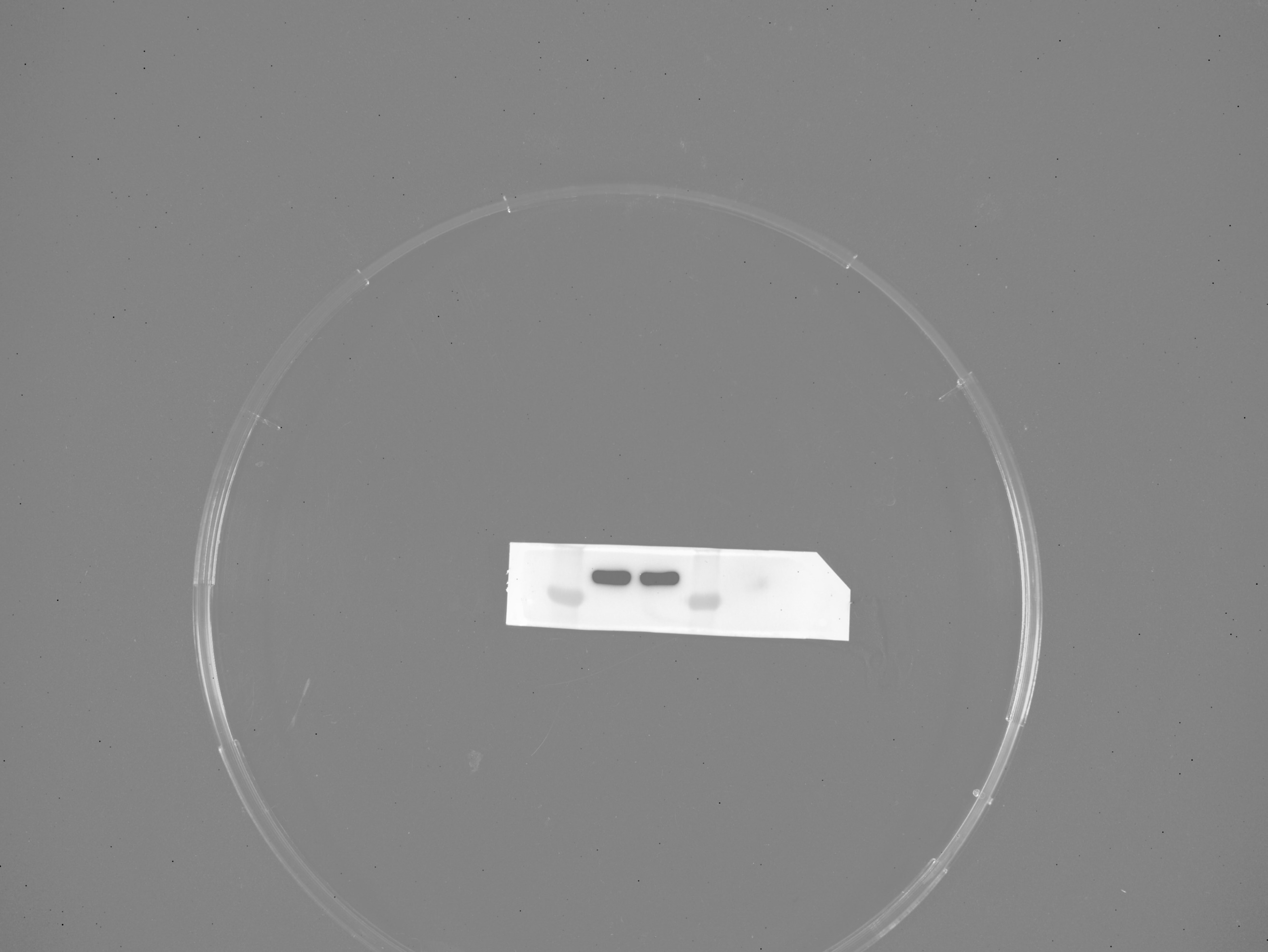


50KDa—

**AKT (60 KDa)**


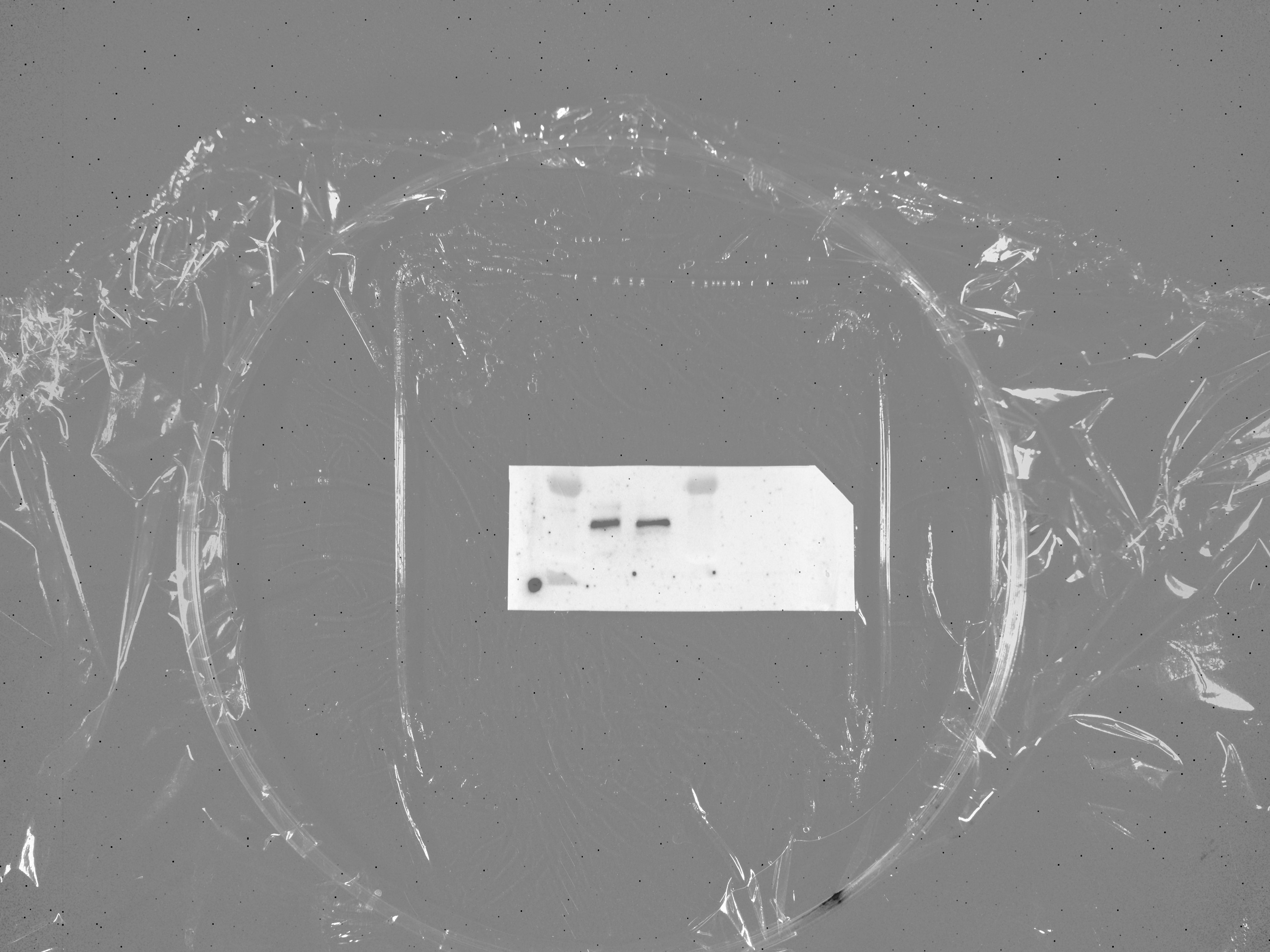


37KDa—

**IGFBP4 (34 KDa)**


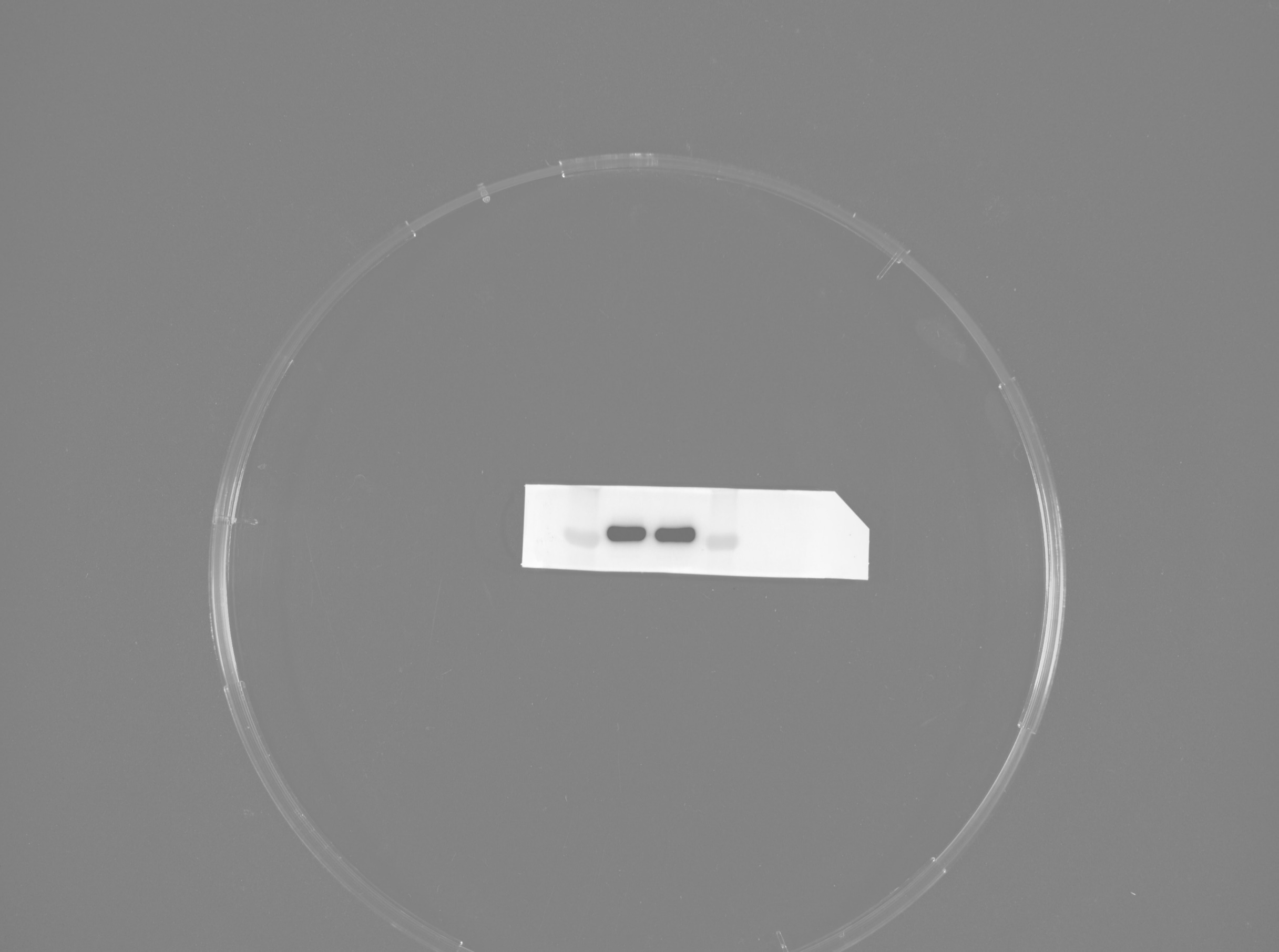


**(D)**

**(B)**

50KDa—

**α-tubulin (50 KDa)**


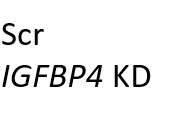

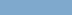

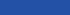


**(C)**

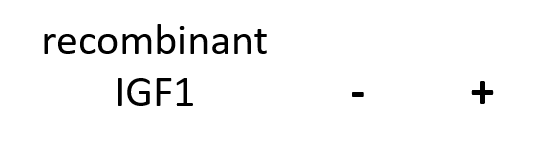

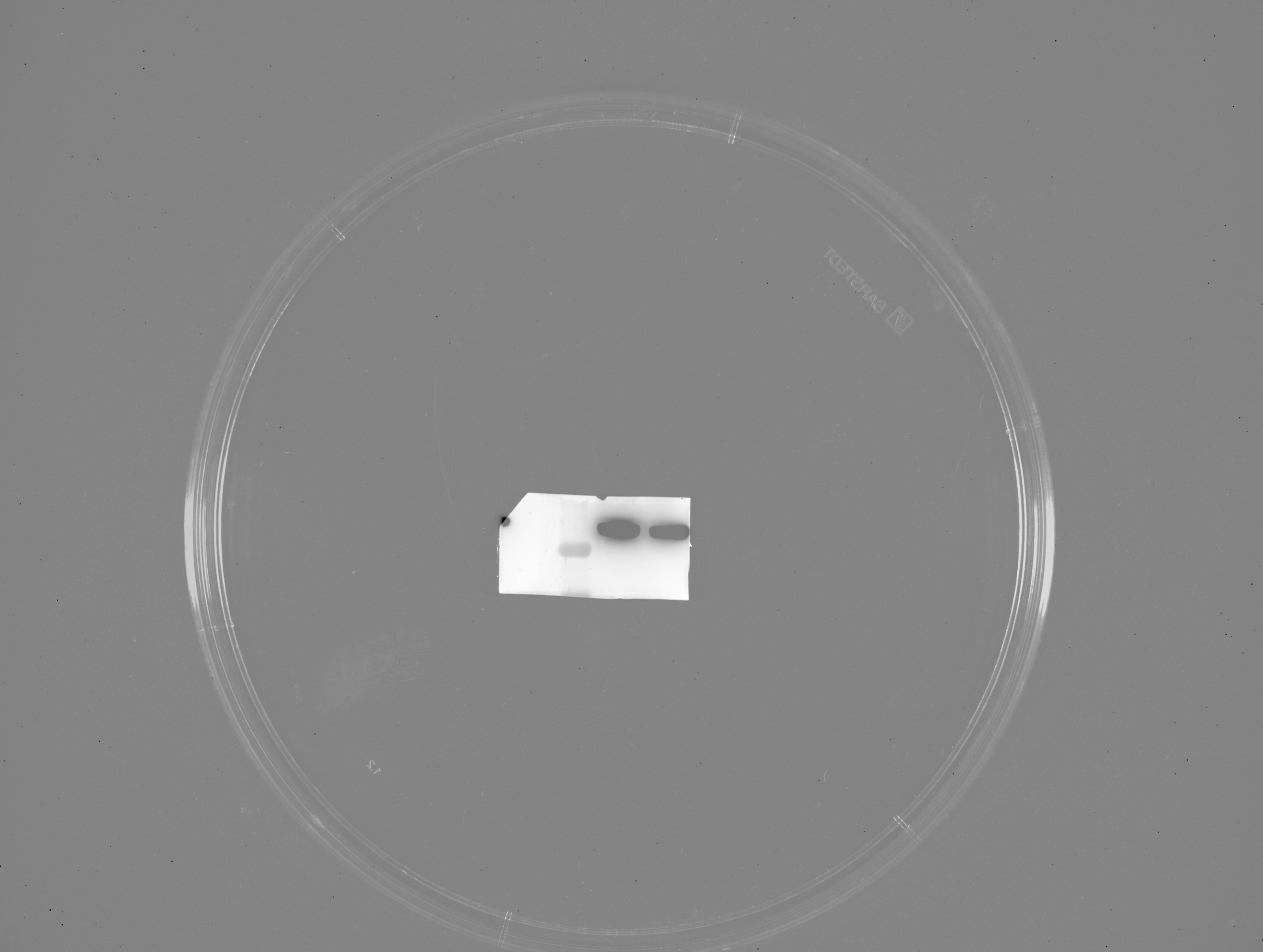

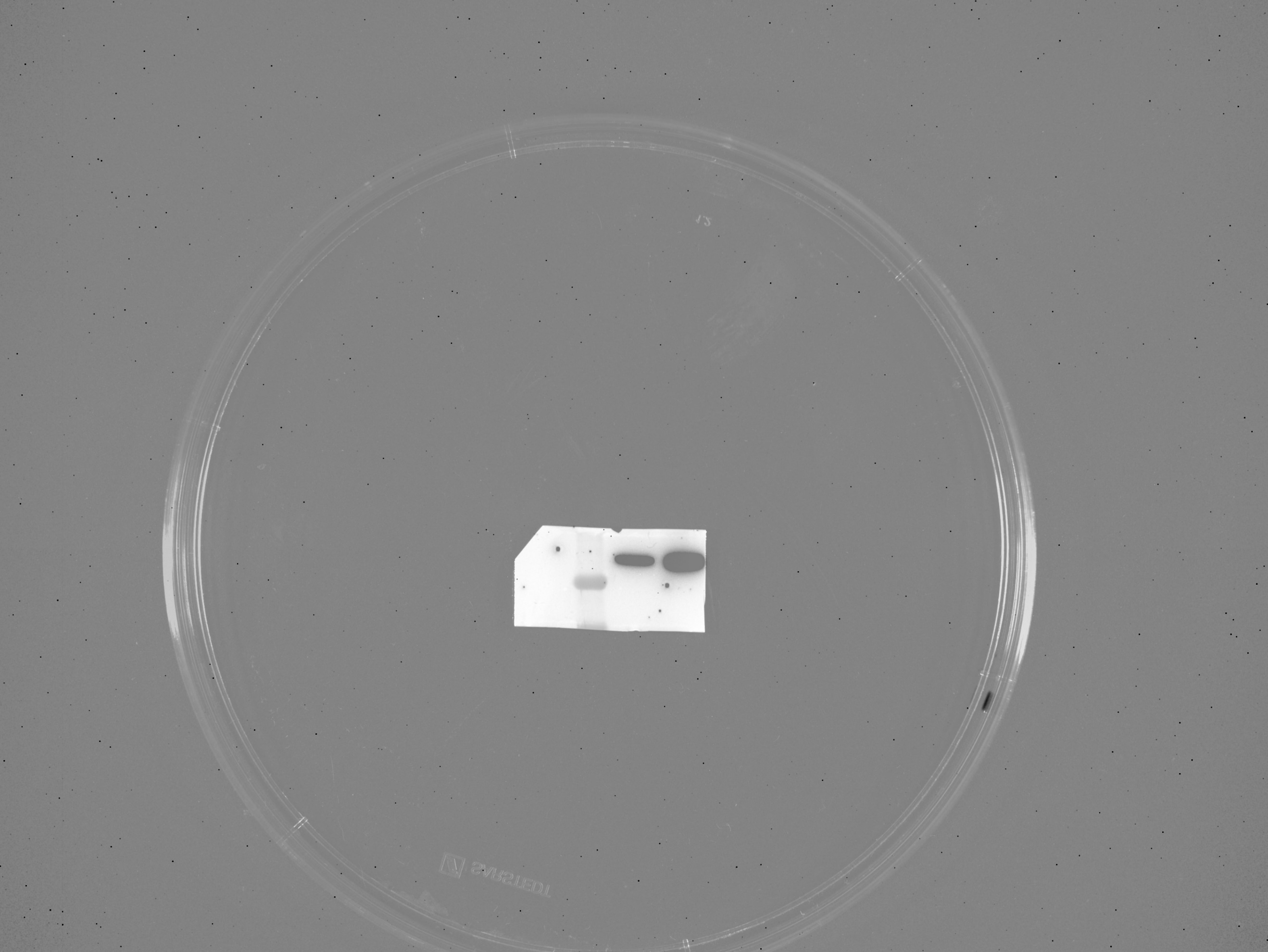

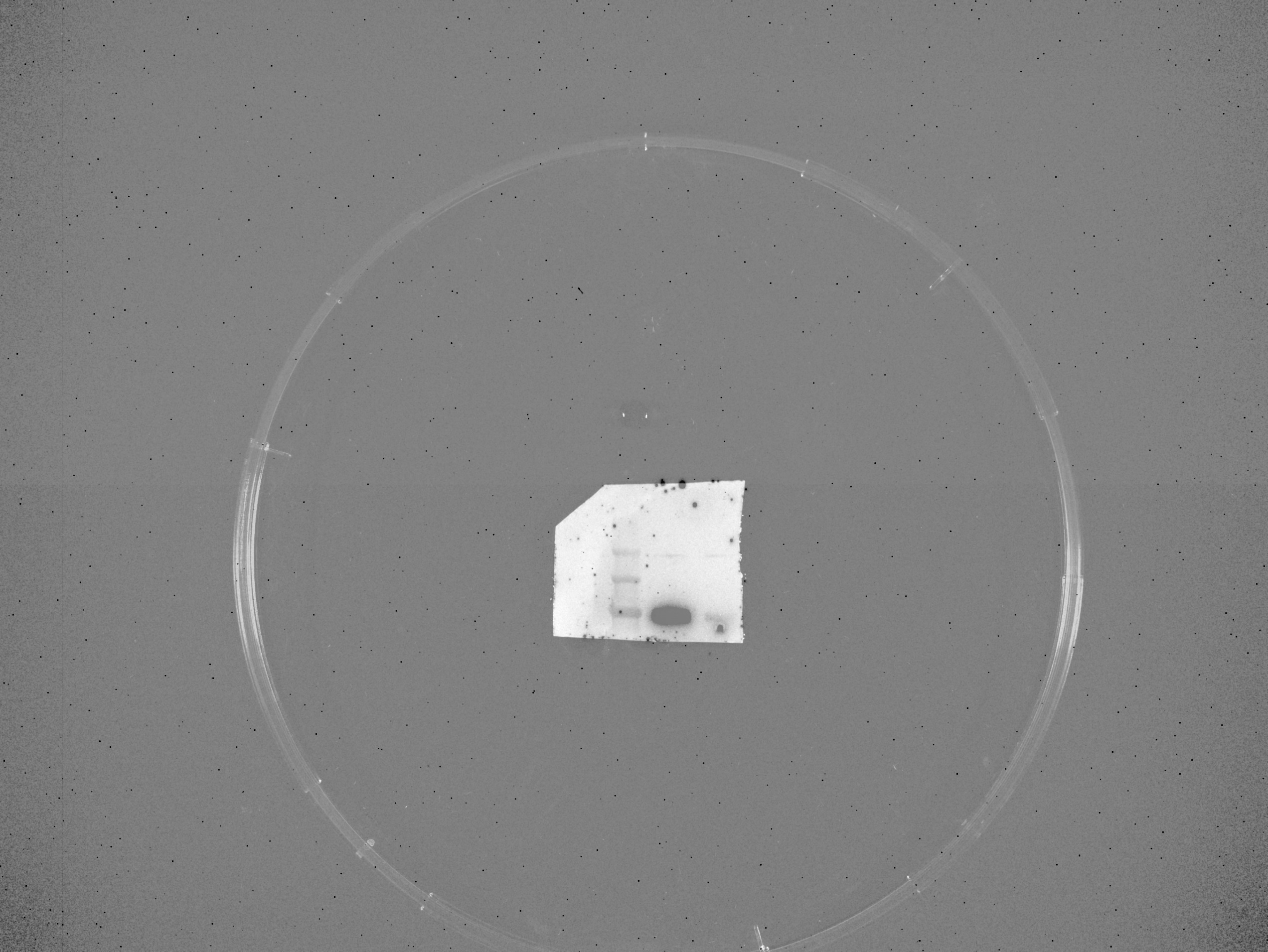

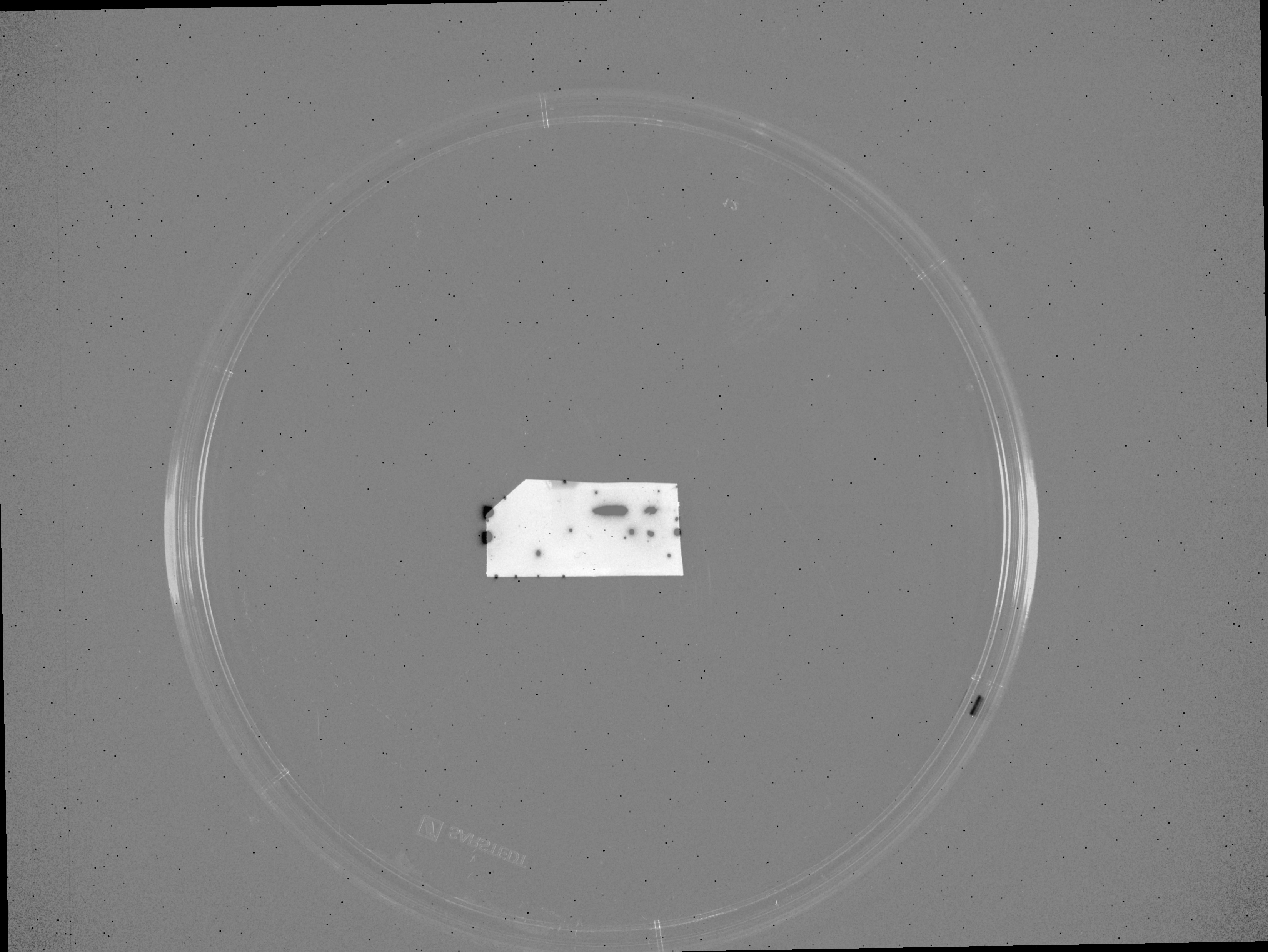

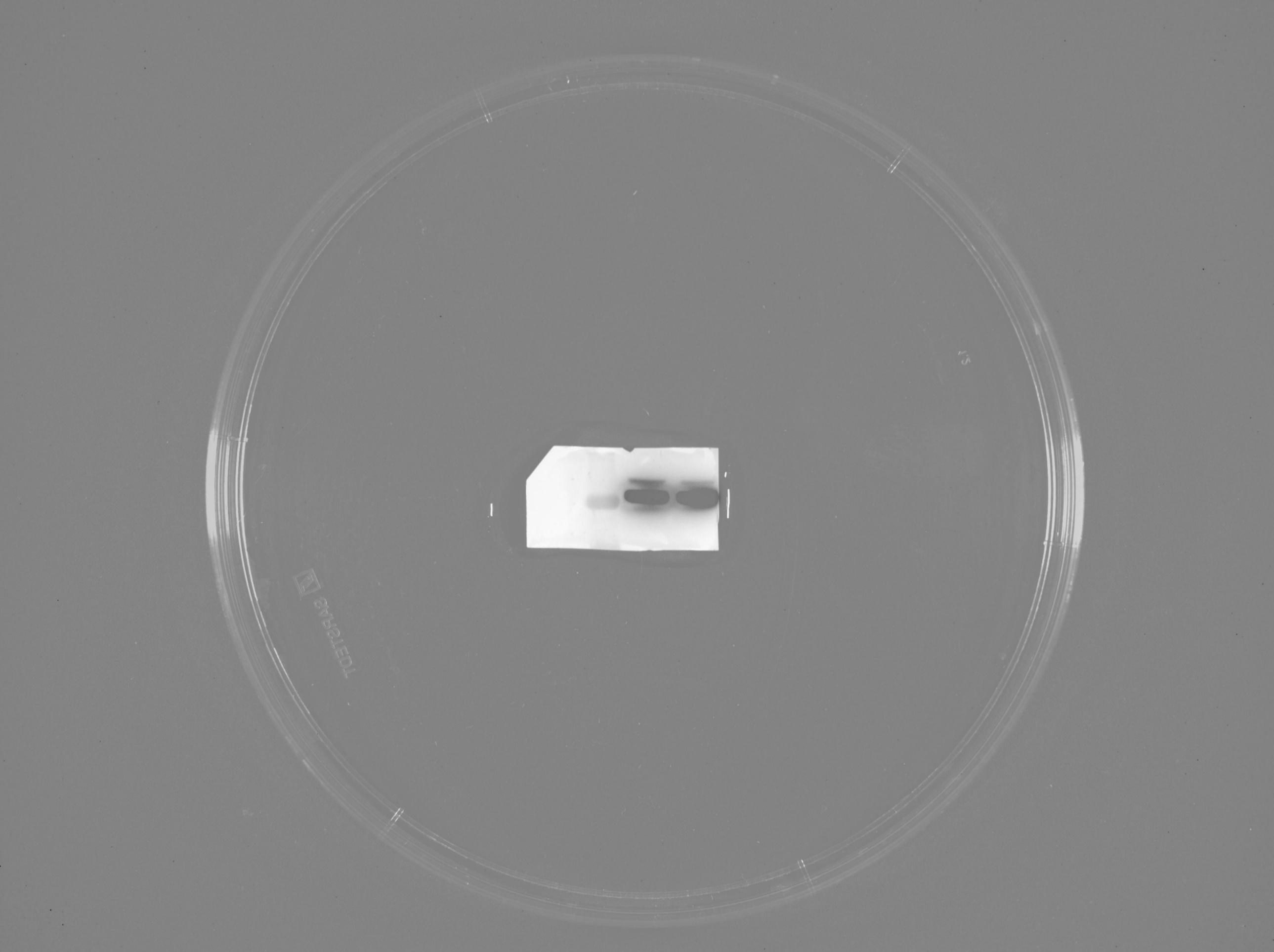


**(D)**

**IGF1R (95 KDa)**

**pAKT (Ser473)**

**AKT (60 KDa)**

**IGFBP4 (34 KDa)**

**α-tubulin (50 KDa)**

100KDa—

50KDa—

50KDa—

37KDa—

**(C)**

**Supplementary Figure 6: Effect of adding recombinant IGF1 on IGF1R and phosphorylated AKT (pAKT, Ser473)**

Representative Western blot images (A) and densitometric analysis (B) of adding recombinant IGF1 (1 µM) to the culture medium. It showed decreased expression of IGF1R and IGFBP4 protein but higher phosphorylated AKT (Ser473). Data were presented as mean ± SD (n = 2).

**(B)**

**(A)**

50KDa—
